# Parathyroid hormone and phosphate homeostasis in patients with Bartter and Gitelman syndrome: an international cross-sectional study

**DOI:** 10.1093/ndt/gfac029

**Published:** 2022-02-07

**Authors:** Maartje F A Verploegen, Rosa Vargas-Poussou, Stephen B Walsh, Harika Alpay, Atefeh Amouzegar, Gema Ariceta, Bahriye Atmis, Justine Bacchetta, Peter Bárány, Stéphanie Baron, Umut Selda Bayrakci, Hendrica Belge, Martine Besouw, Anne Blanchard, Arend Bökenkamp, Olivia Boyer, Kathrin Burgmaier, Lorenzo A Calò, Stéphane Decramer, Olivier Devuyst, Maria van Dyck, Pietro Manuel Ferraro, Marc Fila, Telma Francisco, Gian Marco Ghiggeri, Leire Gondra, Stefano Guarino, Nakysa Hooman, Ewout J Hoorn, Pascal Houillier, Konstantinos Kamperis, Jameela A Kari, Martin Konrad, Elena Levtchenko, Laura Lucchetti, Francesca Lugani, Pierluigi Marzuillo, Barian Mohidin, Thomas J Neuhaus, Abdaldafae Osman, Svetlana Papizh, Manel Perelló, Maarten B Rookmaaker, Valerie Said Conti, Fernando Santos, Ghalia Sawaf, Erkin Serdaroglu, Maria Szczepanska, Francesca Taroni, Rezan Topaloglu, Francesco Trepiccione, Enrico Vidal, Elizabeth R Wan, Lutz Weber, Zeynep Yuruk Yildirim, Selçuk Yüksel, Galia Zlatanova, Detlef Bockenhauer, Francesco Emma, Tom Nijenhuis

**Affiliations:** Department of Nephrology, Radboud University Medical Center, Nijmegen, The Netherlands; Department of Genetics, Centre de Références MARHEA, Hôpital Européen Georges Pompidou Assistance Publique Hôpitaux de Paris, Paris, France; Department of Renal Medicine, University College London, London, UK; Division of Paediatric Nephrology, Faculty of Medicine, Marmara University, Istanbul, Turkey; Division of Nephrology, Department of Medicine, Firoozgar Clinical Research Development Center, Iran University of Medical Sciences, Tehran, Iran; Paediatric Nephrology Department, University Hospital Vall d'Hebron, Barcelona, Spain; Department of Paediatric Nephrology, Cukurova University Faculty of Medicine, Adana, Turkey; Department of Paediatric Nephrology, Rheumatology and Dermatology, Reference Centre for Rare Renal Diseases, Reference Centre for Rare Diseases of Calcium and Phosphate Metabolism. University Children's Hospital, Lyon, France; Division of Renal Medicine, Department of Clinical Science, Intervention and Technology, Karolinska Institutet, Karolinska University Hospital, Stockholm, Sweden; Department of Physiology, Hôpital Européen Georges Pompidou Assistance Publique Hôpitaux de Paris, Paris, France; Department of Paediatric Nephrology, Ankara City Hospital, Üniversiteler Mahallesi Bilkent Caddesi, Çankaya/Ankara, Turkey; Center for Human Genetics, Institute of Pathology and Genetics, Gosselies, Belgium; Department of Paediatric Nephrology, University of Groningen, University Medical Centre Groningen, Groningen, The Netherlands; Clinical Research Centre 1418, Centre de Références MARHEA, Hôpital Européen Georges Pompidou Assistance Publique Hôpitaux de Paris, Paris, France; Centre de Recherche des Cordeliers, INSERM, Sorbonne Université, Université de Paris, Paris, France; Department of Paediatric Nephrology, Emma Children's Hospital, Amsterdam UMC, Vrije Universiteit Amsterdam, Amsterdam, The Netherlands; Department of Paediatric Nephrology, Necker Hospital, APHP, MARHEA, Imagine Institute, Paris University, Paris, France; Faculty of Medicine, University Hospital of Cologne, Children's and Adolescents' Hospital, Paediatric Nephrology, University of Cologne, Cologne, Germany; Department of Medicine (DIMED), Nephrology, Dialysis, Transplantation, University of Padova, Padova, Italy; Department of Paediatric Nephrology. Centre de Références SORARE, Toulouse University Hospital, Toulouse, France; Division of Nephrology, UCLouvain Medical School, Brussels, Belgium; Institute of Physiology, Mechanism of Inherited Kidney Disorders Group, University of Zurich, Zurich, Switzerland; Department of Paediatric Nephrology, University Hospital Leuven, Leuven, Belgium; U.O.S. Terapia Conservativa della Malattia Renale Cronica, U.O.C. Nefrologia, Fondazione Policlinico Universitario A. Gemelli IRCCS, Rome, Italy; Dipartimento Universitario di Medicina e Chirurgia Traslazionale, Università Cattolica del Sacro Cuore, Rome, Italy; Pediatric Nephrology, CHU Arnaud de Villeneuve, Montpellier University Hospital, Montpellier, France; Department of Paediatric Nephrology, Centro Hospitalar Universitário de Lisboa Central, Lisbon, Portugal; Division of Nephrology, Dialysis and Transplantation, Istituto Giannina Gaslini, IRCCS, Genoa, Italy; Pediatric Nephrology Department, Cruces University Hospital, Barakaldo, Spain; Biocruces Bizkaia Health Research Institute, Barakaldo, Spain; Paediatric Department, University of the Basque Country UPV/EHU, Leioa, Spain; Department of Woman, Child and of General and Specialized Surgery, Università degli Studi della Campania ‘Luigi Vanvitelli’, Naples, Italy; Ali-Asghar Clinical Research Development Centre, Iran University of Medical Sciences, Tehran, Iran; Department of Internal Medicine, Division of Nephrology and Transplantation, Erasmus Medical Centre, University Medical Centre Rotterdam, Rotterdam, The Netherlands; Centre de Recherche des Cordeliers, INSERM, Sorbonne Université, Université de Paris, Paris, France; Department of Physiology, Centre de Références MARHEA, Hôpital Européen Georges Pompidou Assistance Publique Hôpitaux de Paris, Paris, France; Department of Paediatrics and Adolescent Medicine, Aarhus University Hospital, Aarhus, Denmark; Pediatric Nephrology Centre of Excellence and Paediatric Department, Faculty of Medicine, King Abdulaziz University, Jeddah, Kingdom of Saudi Arabia; Department of General Paediatrics, Paediatric Nephrology, University Hospital Münster, Munster, Germany; Department of Paediatric Nephrology & Department of Development and Regeneration, University Hospital Leuven, KU Leuven, Leuven, Belgium; Department of Paediatric Subspecialties, Division of Nephrology, Bambino Gesù Children's Hospital – IRCCS, Rome, Italy; Division of Nephrology, Dialysis and Transplantation, Istituto Giannina Gaslini, IRCCS, Genoa, Italy; Department of Woman, Child and of General and Specialized Surgery, Università degli Studi della Campania ‘Luigi Vanvitelli’, Naples, Italy; Department of Renal Medicine, University College London, London, UK; Department of Paediatrics, Children's Hospital of Lucerne, Cantonal Hospital of Lucerne, Lucerne, Switzerland; Paediatric Nephrology Unit, Great Ormond Street Hospital, London, UK; Department of Hereditary and Acquired Kidney Diseases, Research and Clinical Institute for Pediatrics, Pirogov Russian National Research Medical University, Moscow, Russia; Nephrology Department, Hospital Universitari Vall d'Hebron, Universitat Autònoma de Barcelona, Barcelona, Spain; Department of Nephrology and Hypertension, University Medical Centre Utrecht, Utrecht, The Netherlands; Department of Paediatrics, Mater Dei Hospital Malta, Msida, Malta; Department of Paediatrics, Asturias Central University Hospital, University of Oviedo, Oviedo, Spain; Department of Paediatric Nephrology, Damascus Hospital, Damascus, Syria; Department of Paediatric Nephrology, Dr Behçet Uz Children's Hospital, Izmir, Turkey; Department of Paediatrics, Faculty of Medical Sciences in Zabrze, SUM in Katowice, Katowice, Poland; Paediatric Nephrology, Dialysis and Transplant Unit, Fondazione IRCCS Ca’ Granda-Ospedale Maggiore Policlinico, Milan, Italy; Department of Paediatric Nephrology, Hacettepe University School of Medicine, Ankara, Turkey; Department of Translational Medical Sciences, University of Campania “L. Vanvitelli”, Naples, Italy; Division of Paediatrics, Department of Medicine, University of Udine, Udine, Italy; Department of Renal Medicine, University College London, London, UK; Faculty of Medicine, University Hospital of Cologne, Children's and Adolescents' Hospital, Paediatric Nephrology, University of Cologne, Cologne, Germany; Department of Paediatrics, Division of Paediatric Nephrology, Istanbul University Istanbul Faculty of Medicine, Istanbul, Turkey; Department of Paediatric Nephrology, Pamukkale University School of Medicine, Denizli, Turkey; University Children's Hospital Medical University, Sofia, Bulgaria; Department of Renal Medicine, University College London, London, UK; Paediatric Nephrology Unit, Great Ormond Street Hospital, London, UK; Department of Paediatric Subspecialties, Division of Nephrology, Bambino Gesù Children's Hospital – IRCCS, Rome, Italy; Department of Nephrology, Radboud University Medical Center, Nijmegen, The Netherlands

**Keywords:** Bartter syndrome, Gitelman syndrome, parathyroid hormone, phosphate, salt losing tubulopathies

## Abstract

**Background:**

Small cohort studies have reported high parathyroid hormone (PTH) levels in patients with Bartter syndrome and lower serum phosphate levels have anecdotally been reported in patients with Gitelman syndrome. In this cross-sectional study, we assessed PTH and phosphate homeostasis in a large cohort of patients with salt-losing tubulopathies.

**Methods:**

Clinical and laboratory data of 589 patients with Bartter and Gitelman syndrome were provided by members of the European Rare Kidney Diseases Reference Network (ERKNet) and the European Society for Paediatric Nephrology (ESPN).

**Results:**

A total of 285 patients with Bartter syndrome and 304 patients with Gitelman syndrome were included for analysis. Patients with Bartter syndrome type I and II had the highest median PTH level (7.5 pmol/L) and 56% had hyperparathyroidism (PTH >7.0 pmol/L). Serum calcium was slightly lower in Bartter syndrome type I and II patients with hyperparathyroidism (2.42 versus 2.49 mmol/L; *P* = .038) compared to those with normal PTH levels and correlated inversely with PTH (*r*_s_ −0.253; *P* = .009). Serum phosphate and urinary phosphate excretion did not correlate with PTH. Overall, 22% of patients had low serum phosphate levels (phosphate—standard deviation score < −2), with the highest prevalence in patients with Bartter syndrome type III (32%). Serum phosphate correlated with tubular maximum reabsorption of phosphate/glomerular filtration rate (TmP/GFR) (*r*_s_ 0.699; *P* < .001), suggesting renal phosphate wasting.

**Conclusions:**

Hyperparathyroidism is frequent in patients with Bartter syndrome type I and II. Low serum phosphate is observed in a significant number of patients with Bartter and Gitelman syndrome and appears associated with renal phosphate wasting.

## Graphical Abstract

**Graphical Abstract:**
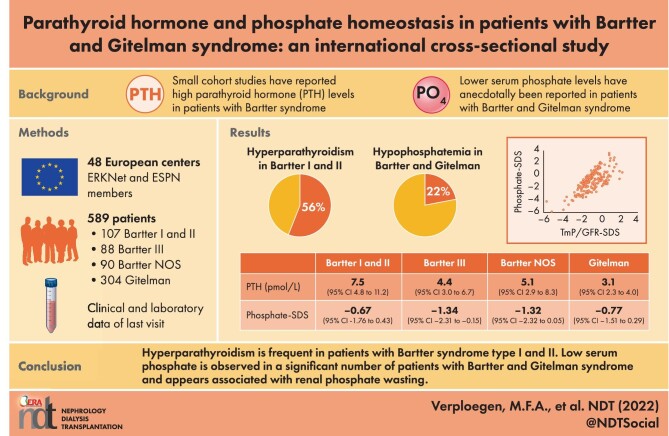

KEY LEARNING POINTS **What is already known about this subject?**

– High parathyroid hormone (PTH) levels have been observed in patients with Bartter syndrome in small cohort studies.
– In patients with Gitelman syndrome, low serum phosphate levels have anecdotally been reported.
– Data from large cohort studies on PTH and phosphate homeostasis in Bartter and Gitelman syndrome are lacking.

**What this study adds?**

– Up till now, this is the largest cohort of patients with salt-losing tubulopathies reported, comprising 285 patients with Bartter syndrome and 304 patients with Gitelman syndrome.
– Elevated PTH is frequently observed in patients with Bartter syndrome, especially in type I and II.
– In a significant number of patients with Bartter and Gitelman syndrome low serum phosphate was reported and is most likely related to a primarily PTH-independent renal phosphate leak.

**What impact this may have on practice or policy?**

– This study provides unique insights into the prevalence of aberrant PTH and phosphate homeostasis in patients with Bartter and Gitelman syndrome.
– Further studies are necessary to understand the pathophysiology of these abnormalities and to demonstrate whether PTH and/or phosphate should be a treatment target in these salt-losing tubulopathies.

## INTRODUCTION

Bartter and Gitelman syndrome are genetically distinct hereditary salt-losing tubulopathies that are characterized by hypokalaemic metabolic alkalosis secondary to impaired reabsorption of sodium chloride (NaCl) in different segments of the renal tubule [1, 2]. Bartter syndrome is further classified into different subtypes. Bartter syndrome type I and II are caused by biallelic mutations in the *SLC12A1* gene encoding the NKCC2 sodium-potassium-chloride cotransporter and by biallelic mutations in the *KCNJ1* gene encoding the ROMK potassium channel, respectively, both of which are expressed in the thick ascending limb of the loop of Henle (TAL). Bartter syndrome type III results from biallelic mutations in the *CLCNKB* gene, which encodes the ClC-Kb chloride channel. Bartter syndrome type IV is caused by biallelic mutations in the *BSND* gene that encodes Barttin, or by molecular abnormalities affecting the two adjacent *CLCNKA* (encoding the chloride channel ClC-Ka) and *CLCNKB* genes. Barttin and ClC-Kb are expressed in the TAL and distal convoluted tubule (DCT); ClC-Ka is only expressed in the TAL. Finally, a transient X-linked form of Bartter syndrome (type V Bartter syndrome) has been described in male patients harbouring mutations in the *MAGED2* gene, which alters the expression of the NKCC2 and NCC, the sodium chloride cotransporter. Gitelman syndrome is caused by biallelic mutations in the *SLC12A3* gene, encoding the NCC, of which the expression is restricted to the DCT.

While Bartter and Gitelman syndromes share similarities, including hypokalaemia, metabolic alkalosis, hyperreninemic hyperaldosteronism and low/normal blood pressure, differences in the tubular segments where NaCl reabsorption is compromised produce different clinical pictures [2]. Patients with Gitelman syndrome usually present in late childhood or early adulthood. Typically, patients with Gitelman syndrome have hypomagnesemia and hypocalciuria. Conversely, type I and II Bartter syndrome patients usually present antenatally with polyhydramnios and postnatally with severe polyuria, hypercalciuria and early onset nephrocalcinosis. Patients with type III Bartter syndrome have variable phenotypes that, in some cases, are difficult to distinguish from Gitelman syndrome. The large phenotypic spectrum of Bartter III patients is most likely explained by variable expression of the pathogenic gene product in the TAL and DCT and by the possibility of partial compensation by other chloride channels, in particular, ClC-Ka in the TAL.

In recent years, several investigators have observed abnormal levels of parathyroid hormone (PTH) and serum phosphate in patients with Bartter and Gitelman syndrome [3–13]. Patients with Bartter syndrome tend to have a high PTH [3–9]. However, the prevalence and pathophysiology of hyperparathyroidism in these Bartter patients are unclear. Furthermore, data have been in part contradictory since a tendency towards both hyper- and hypophosphatemia has been described in Bartter syndrome [3, 5]. In patients with Gitelman syndrome, high PTH levels have only been described in patients that had parathyroid adenoma [14, 15], while hypophosphatemia has been reported in several small studies [10–13] and has been attributed to renal phosphate wasting [12, 13].

To verify whether these observations from case reports and small series are prevalent in Bartter and Gitelman syndromes or just incidental findings, we analysed PTH and phosphate levels in a very large cross-sectional cohort of patients with Bartter and Gitelman syndrome collected across Europe, including both paediatric and adult subjects.

## MATERIALS AND METHODS

On behalf of the European Rare Kidney Diseases Reference Network (ERKNet) Tubulopathies Working Group and European Society for Paediatric Nephrology (ESPN) Working Group on Inherited Renal Disorders, members of the ERKNet and ESPN were invited to provide retrospective clinical and laboratory data on patients with Bartter and Gitelman syndrome. Patients had either signed an informed consent form, allowing an anonymized transfer of clinical data within the ERKNet network, or consent was obtained according to local regulations and under the responsibility of local investigators. The data collection form included 38 items, of which some were mandatory, in particular those related to kidney function, PTH levels, and calcium-phosphate metabolism (Supplementary data, Table S1). To minimize biases in estimating the prevalence of signs and symptoms, data collection was cross-sectional and centres were asked to provide only information corresponding to the last available patient visit. For example, if a given patient had transiently developed high PTH in the past, this information would not be captured to avoid overestimating the prevalence of hyperparathyroidism.

**FIGURE 1:**
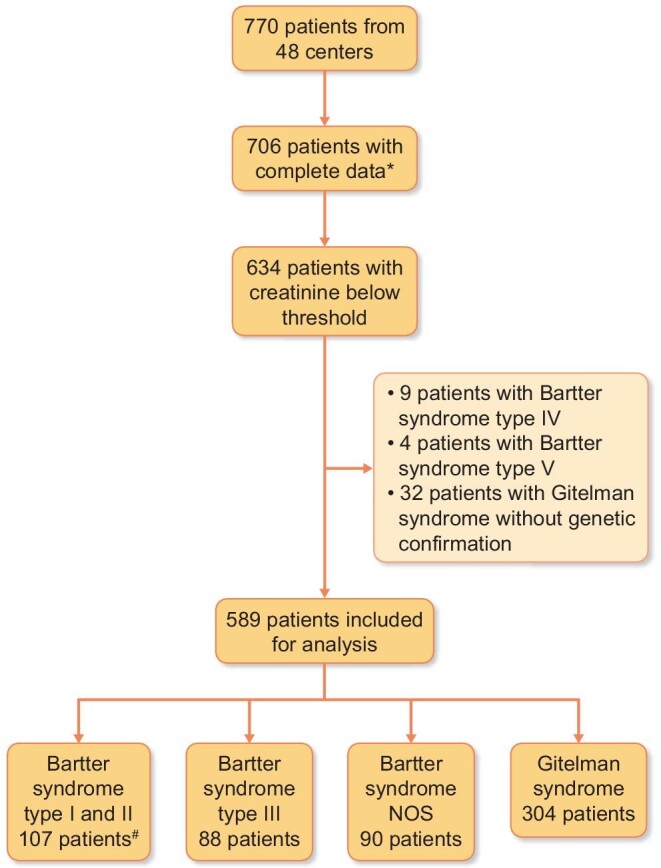
Patient cohort. NOS, not otherwise specified. Patients were grouped for analysis according to the genetic confirmation of disease (subtype). *Creatinine and PTH had to be provided. ^#^ 51 patients with Bartter syndrome type I and 56 patients with Bartter syndrome type II.

Laboratory data were converted to SI units. Outlier data were identified and verified with the referring clinician. Patients with incomplete data on creatinine or PTH were excluded from the analysis. To preclude measuring the effects of low GFR on phosphate and PTH levels, patients with decreased kidney function were excluded. Since estimated GFR (eGFR) data were not available, patients were excluded based on serum creatinine. To this end, we excluded all patients aged >15 years with serum creatinine >100 μmol/L and patients aged ≤15 years with serum creatinine levels above an arbitrary value calculated with the following formula: 2.7 x age (years) + 60. Using these conservative cut-off values, we estimated by preliminary testing that most patients with eGFR < 65 mL/min/1.73m^2^ have been excluded. Overall, we excluded 76 patients with elevated creatinine (Figure 1). A data completeness list is provided in Supplementary data, Table S2.

Serum phosphate, alkaline phosphatase and TmP/GFR are strongly influenced by age in the paediatric population. To be able to compare these variables in all patients, standard deviation scores (SDS) were calculated based on age-related reference values [16–18]. We used the following formula to calculate SDS for phosphate, alkaline phosphatase and TmP/GFR:

$$\begin{equation*}{\rm{SDS}} = \left( {{\rm{level}}-{\rm{mean}}\,\,{\rm{level}}\,\,{\rm{for}}\,\,{\rm{age}}} \right)/{\rm{SD}}\,\,{\rm{for}}\,\,{\rm{age}}.
\end{equation*}$$

Each formula was based on published age-related reference values for phosphate, alkaline phosphatase and TmP/GFR. (Supplementary data and methods, Tables S3 and S4, Figure S1–S3).

Patients were stratified into 4 groups, based on the underlying genetic cause. These include patients with Bartter syndrome type I or II (*n* = 107), Bartter syndrome type III (*n* = 88), Bartter syndrome ‘not otherwise specified’ (NOS) including patients with Bartter syndrome for which genetic data were not available (*n* = 90) and Gitelman syndrome (*n* = 304). Patients with Bartter syndrome type IV (*n* = 9), type V (*n* = 4), or Gitelman syndrome NOS (*n* = 32) were excluded from the final analysis because these tests were severely underpowered (Figure 1).

Data were analysed using IBM SPSS Statistics Version 25. Categorical data are described as numbers and %. Continuous data are presented as median with interquartile range (IQR). Data were analysed using Chi-squared test, Fisher's exact test, Mann–Whitney U test, and Kruskal–Wallis test as appropriate. Spearman's rank correlation was used for the analysis of the correlation between variables. Univariable and multivariable logistic regression were used to identify variables associated with elevated PTH; variables that reached a *P*-value < .10 at the univariable level were used in the multivariable model.

## RESULTS

### Overall characteristics

After excluding subjects with incomplete data, with serum creatinine above the threshold, or underpowered subgroups of patients, 589 patients from the original 770 patients collected were included for analysis (Figure 1).

Patient characteristics are in agreement with the known phenotypic characteristics of the diseases and are detailed in Table 1. The median age was 16.6 years (IQR 8.2–33.5). Patients with Bartter syndrome were significantly younger than patients with Gitelman syndrome [median age 9.5 years (3.5–16.3) versus 28.8 years (16.4–43.9) respectively; *P* < .001). Sex distribution was not significantly different in the four subgroups (*P* = .113). Overall, 52% of the patients were female. Nephrocalcinosis was reported more frequently in patients with Bartter syndrome type I and II compared to type III, with a prevalence of 89 and 18% respectively and very rarely in patients with Gitelman syndrome, as expected. On average, patients with Bartter syndrome had higher urinary calcium/creatinine ratios than Gitelman patients (*P* < .001) and patients with Gitelman syndrome had lower serum magnesium levels than patients with Bartter syndrome (*P* < .001). Indomethacin or other nonsteroidal anti-inflammatory drugs (NSAIDs) were prescribed more often to patients with Bartter syndrome than Gitelman syndrome (*P* < .001).

**Table 1.** Patient characteristics

	Bartter syndrome	Bartter syndrome	Bartter syndrome	Gitelman	
	type I and II	type III	NOS	syndrome	Total
Number of patients	107	88	90	304	589
*Characteristics*					
Male: Female ratio, *n* (%)	51: 56 (48: 52)	50: 38 (57: 43)	48: 42 (53: 47)	133: 171 (44: 56)	282: 307 (48: 52)
Age (years)	9.0 [3.5–16.0]	10.4 [3.3–17.2]	9.6 [3.6–16.3]	28.8 [16.4–43.9]	16.6 [8.2–33.5]
Nephrocalcinosis, *n* (%)	90/101 (89)	15/85 (18)	46/82 (56)	4/257 (2)	155/525 (30)
*Laboratory results*					
iPTH (pmol/L)	7.5 [4.8–11.2]	4.4 [3.0–6.7]	5.1 [2.9–8.3]	3.1 [2.3–4.0]	3.8 [2.5–6.6]
Creatinine (umol/L)	55 [37–76]	43 [–62]	48 [–66]	58 [47–71]	55 [41–70]
Sodium (mmol/L)	140 [138–142]	139 [137–141]	139 [137–140]	139 [138–141]	139 [138–141]
Potassium (mmol/L)	3.6 [3.3–3.8]	3.2 [2.8–3.6]	3.4 [3.0–3.9]	3.0 [2.7–3.4]	3.2 [2.8–3.7]
Chloride (mmol/L)	99 [97–101]	97 [92–100]	96 [93–101]	98 [95–100]	98 [95–100]
Bicarbonate (mmol/L)	26.2 [24.3–28.1]	29.0 [26.3–32.0]	28.4 [27.0–30.7]	29.0 [27.0–31.0]	28.3 [26.3–31.0]
Phosphate (mmol/L)	1.42 [1.21–1.63]	1.28 [1.09–1.44]	1.30 [1.12–1.53]	1.07 [0.92–1.25]	1.21 [0.99–1.42]
Phosphate-SDS	-0.67 [-1.76–0.43]	−1.34 [-2.31– −0.15]	−1.32 [−2.32–0.05]	-0.77 [−1.51–0.29]	−0.91 [−1.82–0.18]
Calcium (mmol/L)	2.44 [2.37–2.53]	2.48 [2.40–2.58]	2.50 [2.38–2.60]	2.41 [2.31–2.50]	2.43 [2.34–2.53]
Magnesium (mmol/L)	0.89 [0.80–0.97]	0.77 [0.68–0.85]	0.81 [0.75–0.95]	0.63 [0.56–0.71]	0.71 [0.61–0.84]
Uric acid (µmol/l)	315 [246–405]	220 [162–319]	286 [190–409]	250 [190–321]	262 [190–350]
Alkaline phosphatase (IU/L)	227 [161–287]	212 [114–274]	230 [160–303]	66 [51–136]	148 [62–245]
Alkaline phosphatase-SDS	0.79 [−0.05–1.52]	0.21 [−0.50–0.99]	0.65 [−0.36–1.89]	−1.00 [−1.72–0.00]	−0.13 [−1.18–0.93]
Total protein (g/L)	73 [70–77]	73 [69–76]	75 [71–78]	73 [69–76]	73 [70–77]
Albumin (g/L)	46 [43–49]	45 [43–48]	46 [43–49]	46 [44–49]	46 [43–49]
25OH vitamin D (nmol/L)	39 [–68]	40 [–82]	43 [–64]	61 [–85]	51 [–80]
Urinary calcium/creatinine (mmol/mmol)	1.55 [0.94–2.62]	0.33 [0.14–0.71]	0.55 [0.23–1.14]	0.08 [0.04–0.17]	0.21 [0.07–0.75]
TRP	0.90 [0.81–0.92]	0.90 [0.86–0.93]	0.88 [0.82–0.95]	0.91 [0.86–0.94]	0.90 [0.85–0.94]
TmP/GFR (mmol/L)	1.41 [1.10–1.48]	1.17 [1.02–1.33]	1.11 [0.84–1.35]	0.95 [0.82–1.10]	1.04 [0.84–1.23]
TmP/GFR-SDS	0.23 [−0.46–0.78]	−0.62 [−1.48–0.44]	−0.66 [−2.03–0.14]	−0.98 [−1.47– −0.46]	−0.86 [−1.54– −0.02]
*Treatment*					
NSAIDs, *n* (%)	69 (65)	52 (59)	55 (61)	26 (9)	202 (34)
Indomethacin, *n* (%)	53 (50)	48 (55)	54 (60)	26 (9)	181 (31)
Ibuprophen, *n* (%)	16 (15)	4 (5)	2 (2)	0 (0)	22 (4)
Other NSAIDs, *n* (%)	5 (5)	7 (8)	4 (4)	1 (0.3)	17 (3)
Potassium-sparing diuretics, *n* (%)	2 (2)	12 (14)	4 (4)	63 (21)	81 (14)
Amilorid, *n* (%)	2 (2)	12 (14)	4 (4)	58 (19)	76 (13)
Triamterene, *n* (%)	0 (0)	0 (0)	0 (0)	5 (2)	5 (1)
Aldosterone antagonists, *n* (%)	5 (5)	17 (19)	23 (26)	54 (18)	99 (17)
Eplerenone, *n* (%)	1 (1)	2 (2)	6 (7)	15 (5)	24 (4)
Spironolactone, *n* (%)	4 (4)	15 (17)	16 (18)	37 (12)	72 (12)
Canrenone, *n* (%)	0 (0)	0 (0)	1 (1)	2 (1)	3 (1)
ACE inhibitors/ARBs, *n* (%)	4 (4)	5 (6)	0 (0)	6 (2)	15 (3)
Hydrochlorothiazide, *n* (%)	3 (3)	0 (0)	6 (7)	0 (0)	9 (2)
Potassium supplements, *n* (%)	71 (66)	83 (94)	76 (84)	280 (92)	510 (87)
Sodium supplements, *n* (%)	28 (26)	44 (50)	21 (23)	43 (14)	136 (23)
Magnesium supplements, *n* (%)	11 (10)	21 (24)	18 (20)	226 (74)	276 (47)
Phosphate supplements, *n* (%)	0 (0)	2 (2)	3 (3)	1 (0,3)	6 (1)
Proton pump inhibitors, *n* (%)	20 (19)	24 (27)	14 (16)	39 (13)	97 (17)
Other gastric protectors, *n* (%)	12 (11)	10 (11)	8 (9)	11 (4)	41 (7)
Oral contraceptive, *n* (%)	1 (1)	1 (1)	1 (1)	20 (7)	23 (4)
Vitamin D supplements, *n* (%)	41 (38)	20 (23)	17 (19)	26 (9)	104 (18)

iPTH, intact parathyroid hormone; phosphate-SDS, age-related phosphate standard deviation score; alkaline phosphatase-SDS, age-related alkaline phosphatase standard deviation score; 25OH vitamin D, 25-hydroxy vitamin D; TRP, tubular reabsorption of phosphate; TmP/GFR, ratio of tubular maximum reabsorption of phosphate to GFR; TmP/GFR-SDS: age-related TmP/GFR standard deviation score; NSAIDs, nonsteroidal anti-inflammatory drugs; ACE inhibitors, angiotensin converting enzyme inhibitors; ARBs, angiotensin II receptor blockers.

Categorical values are presented as number and % given in parentheses. Continuous values are presented as median with interquartile range (IQR) given in square brackets.

*Nota bene:* some patients are using more than one NSAID according to the provided data.

**FIGURE 2:**
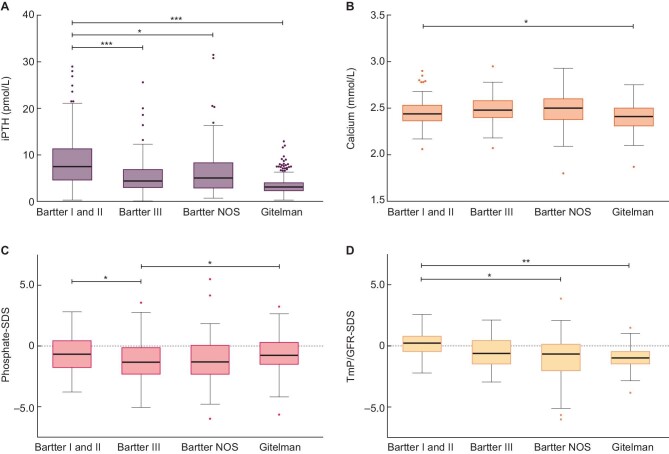
Boxplots of iPTH, calcium, phosphate-SDS and TmP/GFR-SDS according to disease subtype iPTH, intact parathyroid hormone; phosphate-SDS, age-related phosphate standard deviation score; TmP/GFR-SDS, age-related ratio of tubular maximum reabsorption of phosphate to GFR standard deviation score; Boxplot graphs represent the median and IQR; the upper and lower whiskers represent data points within 25th percentile − 1.5x IQR and 75th percentile + 1,5x IQR. Outliers are plotted individually. *P < .05; **P < .01; ***P < .001. **A.** iPTH in pmol/L; statistically significant difference in iPTH level between patients with Bartter syndrome type I and II and patients with Bartter syndrome type III, Bartter syndrome NOS and Gitelman syndrome. **B.** Calcium in mmol/L, statistically significant difference in calcium level between patients with Bartter syndrome type I and II and patients with Gitelman syndrome. **C.** Phosphate-SDS; statistically significant difference in age-adjusted phosphate level between patients with Bartter syndrome type III and patients with Bartter syndrome type I and II and Gitelman syndrome. **D.** TmP/GFR-SDS; statistically significant difference between Bartter syndrome type I and II and Bartter syndrome NOS and Gitelman syndrome.

Elevated PTH levels were observed in a significant proportion of patients. Hyperparathyroidism (iPTH >7.0 pmol/L) was present in 23% of patients (Supplementary data, Table S5). Patients with Bartter syndrome type I and II had the highest median intact parathyroid hormone (iPTH) levels [7.5 pmol/L (4.8–11.2)] (Table 1; Figure 2) and the prevalence of hyperparathyroidism was 56% in this group. Conversely, Gitelman patients had the lowest prevalence of hyperparathyroidism (7%) and even showed a 20% prevalence of hypoparathyroidism (iPTH < 2.0 pmol/L).

All disease subgroups had a negative median phosphate-SDS, demonstrating that median serum phosphate was lower in all subgroups than median age-specific serum phosphate levels (Table 1; Figure 2). Patients with Bartter syndrome type III had the lowest median phosphate-SDS of −1.34 (−2.31 to −0.15), which was significantly lower compared to phosphate-SDS in Bartter syndrome type I and II and Gitelman syndrome (*P* = .032 and *P* = .011 respectively; Figure 2). No differences were observed in tubular reabsorption of phosphate (TRP) (*P* = .254) between different disease subgroups. TmP/GFR-SDS was lowest in patients with Gitelman syndrome [−0.98 (−1.47 to −0.46); Figure 2]. This was significantly lower compared to Bartter syndrome type I and II (*P* = .006).

Median and IQR of serum calcium were within the normal range for all subgroups (Table 1; Figure 2). Serum calcium was significantly higher in patients with Bartter syndrome compared to Gitelman syndrome (*P* < .001). No significant differences in serum calcium were observed between Bartter syndrome subtypes.

Although data were available only in part of the entire cohort (Supplementary data, Table S2), 25-hydroxy vitamin D (25OH vitamin D) levels were lower in patients with Bartter syndrome compared to Gitelman syndrome (*P* = .001) and patients with Bartter syndrome used vitamin D supplements more often (*P* < .001; Table 1). No difference in serum calcium levels was found between patients who used vitamin D supplements and non-supplemented patients (data not shown).

### Hyperparathyroidism and hypophosphatemia

#### Analysis of the entire cohort

The characteristics and laboratory data of patients according to the absence or presence of hyperparathyroidism are shown in Supplementary data, Table S6. Overall, 84% of patients with elevated iPTH had Bartter syndrome. Notably, 45% of the patients with hyperparathyroidism were patients with Bartter syndrome type I and II, while this disease subtype comprises only 18% of patients in the entire cohort. Several parameters, among which urinary calcium/creatinine ratio and serum magnesium, were significantly different when comparing patients with and without hyperparathyroidism (Supplementary data, Table S6) and iPTH was correlated with several parameters (Supplementary data, Table S7). However, these analyses appear markedly influenced by the unequal prevalence of hyperparathyroidism among different disease subgroups, and therefore, may reflect differences related to the underlying disease, rather than differences related to high iPTH. The same limitation applies to the multivariable logistic regression reported in Supplementary data, Table S8 and is underscored by the finding that the strongest predictor for hyperparathyroidism is the Bartter syndrome type I and II sub-class (OR: 3.20, 95% CI 1.68–6.08; *P* < .001). Other significant factors associated with hyperparathyroidism included higher serum magnesium [OR: 18.47 (2.67–127.93) per mmol/L, *P* = .003], lower serum calcium [OR: 0.03 (0.01–0.21) per mmol/L, *P* < .001), higher alkaline phosphatase-SDS [OR: 1.260 (1.11–1.431) per SDS, P < .001] and the use of NSAIDs [OR: 1.88 (1.08–3.28); *P* = .026].

Hypophosphatemia (phosphate-SDS < −2) was observed in 22% of patients (Supplementary data, Table S9). The prevalence of hypophosphatemia was 19% in patients with Bartter syndrome type I and II, 32% in patients with Bartter syndrome type III, and 16% in patients with Gitelman syndrome. Patients with hypophosphatemia had higher serum calcium and lower TmP/GFR-SDS (Supplementary data, Table S10). Phosphate-SDS correlated with TRP (*r*_s_ = 0.201; *P* = .004) and with TmP/GFR-SDS (*r*_s_ = 0.699; *P* < .001) (Supplementary data, Table S11). To exclude an effect of age and/or the necessary modulation of serum phosphate to SDS score to be able to compare paediatric age groups and adults, we also evaluated serum phosphate only in adults. Hypophosphatemia was observed in 14.3% of adult patients (Supplementary data, Table S12). The prevalence of hypophosphatemia was 7% in patients with Bartter syndrome type I and II, 5% in patients with Bartter syndrome type III, and 15% in patients with Gitelman syndrome. However, patient numbers in the Bartter subtype groups were low.

We finally performed a sensitivity analysis, demonstrating that the overall results of Bartter patients were not significantly influenced by the inclusion of the Bartter syndrome NOS cohort (data not shown). Overall, patients included in the NOS subgroup tended to resemble more patients with type I and II Bartter syndrome, especially if they had elevated PTH.

#### Analysis of disease subgroups

When restricting the analysis to patients with Bartter syndrome type I and II, patients with hyperparathyroidism had lower serum calcium levels (2.42 versus 2.49 mmol/L; *P* = .038) and higher alkaline phosphatase-SDS (1.08 versus 0.27; *P* = .010) (Table 2). No differences were observed in serum creatinine, phosphate-SDS, 25OH vitamin D, TmP/GFR-SDS, urinary calcium/creatinine ratio, or the prevalence of nephrocalcinosis. No differences were observed in the prescription of NSAIDs or vitamin D supplements between patients with and without hyperparathyroidism.

**Table 2.** Characteristics of patients with Bartter syndrome type I and II with and without hyperparathyroidism

Variable	Hyperparathyroidisim	No hyperparathyroidism	*P*-value
Number of patients, *n* (%)	60 (56)	47 (44)	
*Characteristics*			
Age (years)	9.6 [3.8–16.3]	8.9 [2.7–15.5]	0.476
Sex (male)	27 (45)	24 (51)	0.563
Nephrocalcinosis, *n* (%)	49/57 (86)	41/44 (93)	0.340
*Laboratory results*			
iPTH (pmol/L)	10.7 [8.6–14.0]	4.5 [3.2–5.6]	NT
Creatinine (µmol L)	54 [–71]	55 [37–77]	0.863
Sodium (mmol/L)	140 [138–142]	140 [138–143]	0.838
Potassium (mmol/L)	3.6 [3.2–3.8]	3.6 [3.3–3.9]	0.538
Chloride (mmol/L)	99 [97–102]	99 [97–101]	0.390
Bicarbonate (mmol/L)	26.0 [24.0–28.0]	26.4 [24.8–28.8]	0.414
Phosphate-SDS	−0.66 [−1.56–0.47]	−0.82 [−1.94–0.21]	0.418
Calcium (mmol/L)	2.42 [2.34–2.51]	2.49 [2.39–2.60]	0.038
Magnesium (mmol/L)	0.90 [0.81–0.98]	0.89 [0.79–0.98]	0.651
Alkaline phosphatase-SDS	1.08 [0.17–1.89]	0.27 [−0.28–1.09]	0.010
Uric acid (µmol/L)	315 [240–403]	327 [262–421]	0.697
Total protein (g/L)	74 [72–78]	72 [68–75]	0.147
Albumin (g/L)	46 [43–48]	47 [43–50]	0.321
25OH vitamin D (nmol/L)	40 [24.5–62.9]	35 [–94]	0.761
Urinary calcium/creatinine (mmol/mmol)	1.46 [0.91–2.42]	1.73 [1.02–2.76]	0.452
TRP	0.87 [0.79–0.93]	0.91 [0.81–0.93]	0.673
TmP/GFR-SDS	−0.13 [−1.26–0.67]	0.56 [−0.36–0.90]	0.481
*Treatment*			
Indomethacin or other NSAID, *n* (%)	37 (62)	32 (68)	0.545
Potassium-sparing diuretics, *n* (%)	2 (3)	0 (0)	0.503
Aldosterone antagonists, *n* (%)	2 (3)	3 (6)	0.652
ACE inhibitors/ARBs, *n* (%)	2 (3)	2 (4)	1.000
Hydrochlorothiazide, *n* (%)	2 (3)	1 (2)	1.000
Potassium supplements, *n* (%)	42 (70)	29 (62)	0.413
Sodium supplements, *n* (%)	17 (28)	11 (23)	0.660
Magnesium supplements, *n* (%)	9 (15)	2 (4)	0.108
Phosphate supplements, *n* (%)	0 (0)	0 (0)	N/A
Proton pump inhibitors, *n* (%)	10 (17)	10 (21)	0.621
Other gastric protectors, *n* (%)	5 (8)	7 (15)	0.360
Oral contraceptives, *n* (%)	1 (2)	0 (0)	1.000
Vitamin D supplements, *n* (%)	26 (43)	15 (32)	0.238

iPTH, intact parathyroid hormone; phosphate-SDS, age-related phosphate standard deviation score; alkaline phosphatase-SDS, age-related alkaline phosphatase standard deviation score; 25OH vitamin D, 25-hydroxy vitamin D; TRP, tubular reabsorption of phosphate; TmP/GFR-SDS, age-related ratio of tubular maximum reabsorption of phosphate to GFR standard deviation score; NSAID, nonsteroidal anti-inflammatory drug; ACE inhibitors, angiotensin converting enzyme inhibitors; ARBs, angiotensin II receptor blockers; NT, not tested; N/A, not applicable

Categorical data is presented as number and % and was analysed by Fisher's exact test. Continuous data is presented as median and interquartile range and was analysed by Mann–Whitney U test.

**FIGURE 3:**
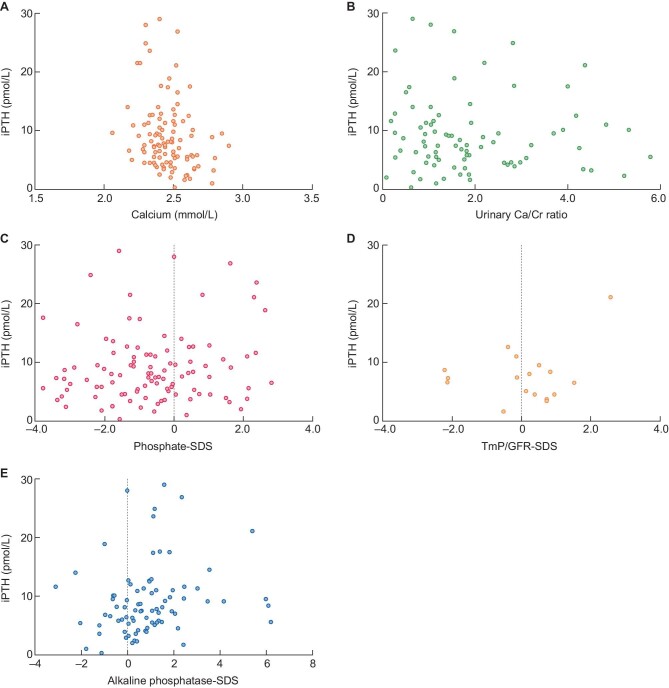
Scatterplots of calcium, urinary calcium/creatinine ratio, phosphate-SDS, TmP/GFR-SDS and alkaline phosphatase-SDS with iPTH of patients with Bartter syndrome type I and II. iPTH, intact parathyroid hormone; phosphate-SDS, age-related phosphate standard deviation score; alkaline phosphatase-SDS, age-related alkaline phosphatase standard deviation score; TmP/GFR-SDS: age-related ratio of tubular maximum reabsorption of phosphate to GFR standard deviation score; **A.** Scatterplot of calcium with iPTH. **B.** Scatterplot of urinary calcium/creatinine ratio with iPTH. **C.** Scatterplot of phosphate-SDS with iPTH. **D.** Scatterplot of TmP/GFR-SDS with iPTH. **E.** Scatterplot of alkaline phosphatase-SDS with iPTH.

In this cohort of patients with Bartter syndrome type I and II, a correlation was observed between iPTH and alkaline phosphatase-SDS (*r*_s_ 0.268, *P* = .015) and an inverse correlation between iPTH and calcium (*r*_s_ −0.253, *P* = .009) (Table 3; Figure 3). No correlation was observed between iPTH and phosphate-SDS, TmP/GFR-SDS, or urinary calcium/creatinine ratio. By multivariable logistic regression analysis, higher alkaline phosphatase-SDS [OR: 1.54 (1.09–2.19) per SDS, *P* = .015] was associated with high iPTH (Table 4). A trend was observed for lower serum calcium [OR: 0.025 (0.001–1.16) per SDS, *P* = .059], which did not reach statistical significance.

**Table 3.** Correlation coefficients of iPTH in patients with Bartter syndrome type I and II

Variable	N	*r_s_*	*P*-value
Sex (male)	107	−0.056	0.569
Age	107	0.116	0.236
Nephrocalcinosis	101	−0.064	0.526
Creatinine	107	0.171	0.078
Sodium	104	0.003	0.974
Potassium	107	−0.112	0.249
Chloride	98	−0.027	0.792
Bicarbonate	99	0.044	0.663
Phosphate-SDS	104	0.104	0.293
Calcium	105	−0.253	0.009
Magnesium	93	0.014	0.897
Uric acid	56	0.002	0.987
Alkaline phosphatase-SDS	82	0.268	0.015
Total protein	48	0.115	0.435
Albumin	75	−0.126	0.283
25OH vitamin D	60	−0.044	0.739
Urinary calcium/creatinine ratio	82	−0.083	0.459
TRP	17	−0.409	0.103
TmP/GFR-SDS	17	−0.085	0.747
Indomethacin or other NSAID	107	−0.095	0.331
Potassium-sparing diuretics	107	0.137	0.158
Aldosterone antagonists	107	−0.115	0.239
ACE inhibitors/ARBs	107	−0.049	0.619
Hydrochlorothiazide	107	−0.003	0.978
Potassium supplements	107	0.102	0.295
Sodium supplements	107	0.042	0.668
Magnesium supplements	107	0.175	0.072
Proton pump inhibitors	107	0.010	0.918
Other gastric protectors	107	−0.083	0.393
Oral contraceptives	107	0.108	0.266
Vitamin D supplements	107	0.102	0.294

iPTH, intact parathyroid hormone; phosphate-SDS, age-related phosphate standard deviation score; alkaline phosphatase-SDS, age-related alkaline phosphatase standard deviation score; 25OH vitamin D, 25-hydroxy vitamin D; TRP, tubular reabsorption of phosphate; TmP/GFR-SDS, age-related ratio of tubular maximum reabsorption of phosphate to GFR standard deviation score; NSAID, nonsteroidal anti-inflammatory drug; ACE inhibitors, angiotensin converting enzyme inhibitors; ARBs, angiotensin II receptor blockers.

r_s_, Spearman's rank correlation coefficient.

**Table 4.** Multivariable logistic regression of patients with Bartter syndrome type I and II for hyperparathyroidism (PTH >7.0 pmol/L)

Variable	Units	N	OR	95% CI	*P*-value
Calcium	mmol/L	80	0.03	0.001–1.16	0.059
Phosphate-SDS		80	1.29	0.93–1.81	0.128
Alkaline phosphatase-SDS		80	1.54	1.09–2.19	0.015
Indomethacin or other NSAID	Yes	80	1.12	0.38–3.34	0.833
Vitamin D supplements	Yes	80	1.34	0.49–3.66	0.566

Phosphate-SDS, age-related phosphate standard deviation score; alkaline phosphatase-SDS, age-related alkaline phosphatase standard deviation score; NSAID, nonsteroidal anti-inflammatory drug; OR, Odds ratio; 95% CI, 95% confidence interval.

In the Gitelman cohort, we observed a significant correlation between iPTH and serum magnesium (*r*_s_ = 0.191, *P* = .001) and an inverse correlation between iPTH and 25OH vitamin D (*r*_s_ = −0.207, *P* = .004) (Supplementary data, Table S13).

**FIGURE 4:**
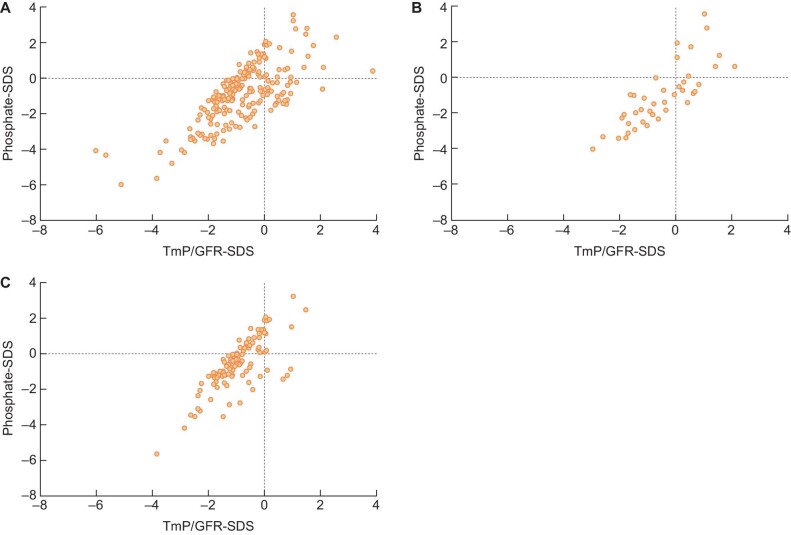
Scatterplots of TmP/GFR with age-adjusted phosphate Phosphate-SDS, age-related phosphate standard deviation score; TmP/GFR-SDS, age-related ratio of tubular maximum reabsorption of phosphate to GFR standard deviation score; **A.** Scatterplot of TmP/GFR with age-adjusted phosphate of all patients. **B.** Scatterplot of TmP/GFR with age-adjusted phosphate of patients with Bartter syndrome type III. **C.** Scatterplots of TmP/GFR with age-adjusted phosphate of patients with Gitelman syndrome.

In addition, as in the entire cohort, a significant correlation was observed between serum phosphate-SDS and TmP/GFR-SDS in patients with Bartter syndrome type III (*r*_s_ = 0.744; *P* < .001) and Gitelman syndrome (*r*_s_ = 0.845; *P* < .001) (Figure 4).

## DISCUSSION

This cross-sectional study analysed PTH and phosphate homeostasis in patients with Bartter and Gitelman syndrome. We observed high PTH (>7.0 pmol/L) in 56% of patients with type I and II disease. All disease subgroups, particularly patients with Bartter syndrome type III, had a tendency towards lower serum phosphate levels and a significant proportion of patients had overt hypophosphatemia (phosphate-SDS < −2).

To date, hyperparathyroidism in these disorders has only been described in case reports and small series [3–9]. For example, Landau *et al.* observed high PTH levels in 10/14 patients with Bartter syndrome type II [5]. Additional small studies did not include genetic characterization [3, 6], while others included only type I and II Bartter syndrome [4, 7–9]. Owing to the large number of patients that were recruited, we were able to analyse subgroups of patients, demonstrating that hyperparathyroidism developed primarily in patients with Bartter syndrome type I and II and to a lesser degree, in patients with Bartter syndrome type III. A major confounding factor when analysing PTH levels is kidney function. To prevent overestimating hyperparathyroidism, we excluded from the analysis all patients with elevated creatinine, using a very conservative threshold. In addition, we observed no correlation between PTH and creatinine levels. We did not observe age-dependent changes in PTH.

The aim of this study was to evaluate the prevalence of hyperparathyroidism and phosphate homeostasis abnormalities in patients with Bartter and Gitelman syndrome. To this end, we designed a cross-sectional study, favouring collection of data on a very large number of patients, over collecting longitudinal data on fewer patients which would have been more informative on pathophysiology, but would have limited our ability to estimate disease prevalence. However, some hypotheses on the pathophysiology and possible clinical implications can be proposed and discussed.

It has previously been hypothesized that hypercalciuria, a characteristic feature of patients with Bartter syndrome type I and type II, may contribute to increased PTH secretion, as a physiological response to maintain serum calcium within the normal range despite excessive renal calcium loss [4, 5, 7, 19]. Indeed, in our whole cohort, a correlation between urinary calcium excretion and PTH was found. Calciuria is a crucial distinction between Bartter and Gitelman syndrome and our observational study cannot distinguish between a causative role of calciuria for hyperparathyroidism or a simple association because of other differences between Bartter and Gitelman syndrome. As expected, hypercalciuria was a consistent feature in patients with Bartter syndrome type I and II, but no correlation between urinary calcium excretion and PTH levels were observed in this subgroup. However, the cross-sectional design of the study and significant heterogeneity of patient treatments, do not allow excluding a possible role of hypercalciuria in promoting PTH secretion. Patients with hyperparathyroidism in this subgroup had on average lower serum calcium levels and, as expected, serum calcium was negatively correlated with PTH. These findings are concordant with the reports by Landau *et al.* and Bettinelli *et al.* [3, 5]. The cause of this lower serum calcium and whether it is sufficient to trigger PTH secretion in these patients remains undetermined.

In our study, high PTH levels in type I and II Bartter syndrome patients were not associated with lower serum phosphate or with higher urinary excretion of phosphate, as also observed by Landau *et al.* [5]. Taken together, these findings raise the possibility of renal hyporesponsiveness to PTH, which could contribute to PTH secretion. Yet, with renal hyporesponsiveness increased serum phosphate could be expected, which we did not observe.

Hyperparathyroidism in Bartter syndrome could also be secondary to prostaglandin secretion [4, 7]. Patients with type I and II disease produce large amounts of prostaglandins due to decreased intracellular NaCl concentration in the macula densa caused by impaired salt reabsorption via the NKCC2 pathway [6, 20, 21]. Treatment of Bartter syndrome patients with NSAIDs reduces renal calcium excretion [19, 20, 22, 23] and in one report, reduces PTH levels [19]. It is unclear in this latter study, if NSAIDs directly inhibit the parathyroid glands, or reduce PTH secretion indirectly, by inhibiting renal calcium excretion. In our study, we did not observe an association between NSAID prescription and PTH levels, nor between NSAID prescription and urinary calcium excretion.

It has also been hypothesized that higher PTH secretion in Bartter syndrome may be secondary to hyperaldosteronism [5]. Parathyroid glands express mineralocorticoid receptors allowing stimulation of PTH secretion by aldosterone [24]. Accordingly, mild hyperparathyroidism has been reported in patients with primary hyperaldosteronism [25] and PTH levels in these patients decrease after adrenalectomy [26]. This hypothesis seems unlikely in patients with salt-losing tubulopathies since patients with Gitelman syndrome, who typically have very high aldosterone levels, do not develop hyperparathyroidism. In addition, PTH levels in our cohort were not associated with treatment with aldosterone antagonists.

Finally, it has been suggested that hypokalaemia could cause hyperparathyroidism and that potassium supplementation could normalize PTH [27]. Here again, we did not observe an inverse correlation between serum potassium and PTH. Moreover, Bartter and Gitelman syndromes are both characterized by hypokalaemia, whereas we observed hyperparathyroidism primarily in patients with Bartter syndrome.

Another noteworthy finding in our cohort was that 20% of patients with Gitelman syndrome had hypoparathyroidism. The calcium-sensing receptor signalling is known to be dependent on extracellular magnesium levels. Unlike calcium, PTH secretion is inhibited by supraphysiologically high magnesium levels via stimulation of the calcium-sensing receptor, but also by low magnesium concentrations [28]. This latter response is suggested to result from intracellular disinhibition of G-protein signalling downstream from the calcium-sensing receptor, precluding PTH release [29]. Accordingly, decreased PTH secretion and decreased responsiveness of PTH to ionized calcium have been suggested in small cohorts of patients with Gitelman syndrome [30, 31, 32]. In agreement with these data, we observed a significant correlation between PTH and serum magnesium in patients with Gitelman syndrome, suggesting that low serum magnesium might be, at least in part, responsible for hypoparathyroidism.

Hypophosphatemia was common in all disease subgroups, in particular in Bartter syndrome type III with a prevalence of 32%. The prevalence of hypophosphatemia in Bartter syndrome type III patients decreased in adulthood to 5%. In Gitelman syndrome, the prevalence of hypophosphatemia was not age-dependent. Low serum phosphate levels were associated with lower TmP/GFR in all subgroups, indicating that hypophosphatemia was most likely related to renal phosphate wasting. However, renal hypophosphatemia did not appear to be driven by hyperparathyroidism, since PTH did not correlate with phosphate-SDS or TmP/GFR-SDS. Furthermore, patients with Gitelman syndrome did not have high PTH levels, while they often had hypophosphatemia and renal phosphate wasting. The exact mechanism(s) of hypophosphatemia in these patients remains unclear. Notably, low serum phosphate levels have already been observed in anecdotal reports on Gitelman syndrome [10–13], including a possible link with lower renal tubular phosphate reabsorption [12, 13]. Interestingly, it has been hypothesized that chronic hypokalaemia can cause proximal tubular cell injury, resulting in a phosphate leak [33]. However, serum potassium and serum phosphate were not correlated in our cohort. Furthermore, FGF23 could play a role. Interestingly, a recent study demonstrated that in the NCC knockout mouse model for Gitelman syndrome, FGF23 levels were increased [34]. In contrast to our Gitelman cohort, these mice also showed increased PTH levels which, however, did not alter fractional phosphate excretion or serum phosphate levels.

We also observed significantly higher serum calcium levels in patients with Bartter syndrome, compared to patients with Gitelman syndrome. Theoretically, since patients with Gitelman syndrome have hypocalciuria, they are expected to have higher serum calcium levels than patients with Bartter syndrome that have hypercalciuria. Furthermore, compared to healthy controls, patients with Gitelman syndrome have higher serum calcium levels [13] and patients with Bartter syndrome tend to have lower serum calcium levels [3]. Vitamin D supplementation may have influenced our results. Although supplements were prescribed more frequently to patients with Bartter syndrome, patients treated with vitamin D did not have significantly higher serum calcium levels, compared to non-supplemented patients.

Whether in clinical practice hyperparathyroidism and hypophosphatemia require further diagnostic evaluation and treatments is unclear. In our cohort, high PTH was associated with higher alkaline phosphatase levels. This is likely due to increased bone turnover. Reduced bone mineral density has previously been observed in patients with Bartter syndrome [35, 36]. It may therefore be appropriate that Bartter syndrome patients undergo a regular assessment of their bone density to detect incipient osteopenia or osteoporosis, particularly if they have high PTH. Since vitamin D deficiency stimulates PTH secretion, supplements could be prescribed in patients with low vitamin D levels, keeping in mind that they may worsen hypercalciuria in patients with Bartter syndrome. Of note, 25-OH vitamin D concentrations are usually normal in patients with Bartter syndrome [23], while high levels of 1,25-OH vitamin D have been reported [23, 36]. The use of calcimimetics in patients with hyperparathyroidism associated with Bartter syndrome has been described in five patients [7, 8]. In four of them, PTH levels normalized. However, all had high-normal or even high serum calcium levels. In theory, normocalcemic patients with Bartter syndrome could be at increased risk of developing symptomatic hypocalcemia if treated with calcimimetics, since they have metabolic alkalosis, which decreases the proportion of ionized calcium. It is unknown whether hypophosphatemia, especially if associated with bone changes, should be treated with phosphate supplementation.

The main limitations of our study relate to the cross-sectional design and the relatively large group of Bartter NOS patients in the analysis. The cross-sectional design allowed us to assemble data on a very large number of patients to estimate the prevalence of PTH and phosphate abnormalities in these diseases. However, longitudinal follow-up of patients would provide additional information about the course of PTH and other variables throughout the years, which could have helped identify possible causal effects. Future studies should collect data at the initial presentation, thus pre-treatment and should also evaluate follow-up data of patients. An important opportunity to prospectively capture such data is by adding PTH and phosphate as variables to the ERKNet patient registry (ERKReg). The second main limitation is the relatively large group of patients with Bartter syndrome NOS, who lacked a genetic diagnosis and were diagnosed based on their phenotype. This subgroup was most likely composed of patients with mixed Bartter syndrome subtypes since their characteristics are intermediate between the type I and II and the type III subgroups. Sensitivity analyses performed removing the NOS subgroup showed that the inclusion of these patients did not modify our results.

Other limitations of our study are related to its retrospective, cross-sectional and multicentric design. The number of variables that could be reliably captured in the database was therefore limited. For example, active vitamin D, ionized calcium, cyclic AMP, FGF-23, aldosterone levels and data on bone metabolism were not available for most patients but could have been very instrumental in understanding the physiopathology of our findings. Treatments were decided by local physicians according to local protocols. Laboratory values were measured in different laboratories, which may have used slightly different methods and reference values. These latter aspects were probably well compensated for by the large size of our cohort. Since the age range of our patients spanned from small children to late adulthood, some values had to be normalized for age using reference data from the literature. This may have introduced some distortions in our data.

In conclusion, this cross-sectional study demonstrates that PTH is frequently elevated in patients with Bartter syndrome, especially in those with type I and II disease. The cause of hyperparathyroidism in this population remains unclear. Hypophosphatemia was observed in a significant proportion of patients with Bartter and Gitelman syndrome and appeared to be primarily related to a PTH-independent renal phosphate leak. Further studies are needed to understand better the pathophysiology and the clinical relevance of these observations.

## Supplementary Material

gfac029_Supplemental_File

## Data Availability

The data underlying this article will be shared on reasonable request to the corresponding author.
