# Supplementary material for: Parathyroid hormone and phosphate homeostasis in patients with Bartter and Gitelman syndrome: an international cross-sectional study
Source: Nephrol Dial Transplant. 2022 Feb 7;37(12):2474–86. doi: 10.1093/ndt/gfac029 (PMC9681919; doi:10.1093/ndt/gfac029)
Supplement: gfac029_Supplemental_File [file gfac029_supplemental_file.pdf]

SUPPLEMENTARY MATERIAL

Supplementary material to: **“**Parathyroid hormone and phosphate homeostasis in patients with Bartter and Gitelman syndrome: an international cross-sectional study”

INDEX

Supplemental methods 2

Formulas for age-related standard deviation score (SDS) of phosphate, alkaline phosphatase and TmP/GFR........ 2

Supplemental tables 6

Supplemental table 1. Items of data collection form 6

Supplemental table 2. Valid variables and missing values 7

Supplemental table 3. Correlation coefficients of age in all patients 9

Supplemental table 4. Correlation coefficients of age in patients with Bartter syndrome type I & II 10

Supplemental table 5. Prevalence of hypo- and hyperparathyroidism 11

Supplemental table 6. Characteristics of all patients with and without hyperparathyroidism 12

Supplemental table 7. Correlation coefficients of iPTH in all patients........ 14

Supplemental table 8. Multivariable logistic regression of hyperparathyroidism in all patients 15

Supplemental table 9. Prevalence of hypo- and hyperphosphatemia 16

Supplemental table 10. Characteristics of all patients with and without hypophosphatemia........ 17

Supplemental table 11. Correlation coefficients of phosphate-SDS in all patients 19

Supplemental table 12. Prevalence of hypo- and hyperphosphatemia in adult patients only........ 20

Supplemental table 13. Correlation coefficients of iPTH in patients with Gitelman syndrome 21

Supplemental figures 22

Supplemental figure 1. Scatterplots of phosphate and phosphate-SDS with age in all patients and patients with Bartter syndrome type I & II 22

Supplemental figure 2. Scatterplots of alkaline phosphatase and alkaline phosphatase-SDS in all patients and patients with Bartter syndrome type I & II 23

Supplemental figure 3. Scatterplots of TmP/GFR and TmP/GFR-SDS in all patients 24

Supplemental methods

Formulas for age-related standard deviation score (SDS) of phosphate, alkaline phosphatase and TmP/GFR

Based on published age-related reference intervals of phosphate, alkaline phosphatase, and TmP/GFR, formulas were created to calculate a normal reference mean value, standard deviation (SD), and upper and lower limits (16-18). We used these continuous reference values instead of reference data of large age intervals for the calculation of the SDS of phosphate, alkaline phosphatase, and TmP/GFR.

<u>Phosphate:</u>

Two different age-related reference intervals were used for phosphate data:

Mean = A1*exp(-x/t1) + y1
SD = [A1*exp(-x/t1) - A2*exp(-x/t2) + y1 - y2]/2

Y1	0.0	Y2	0.206158
A1	2.080034	A2	1.464966
t1	30.29102	T2	22.265

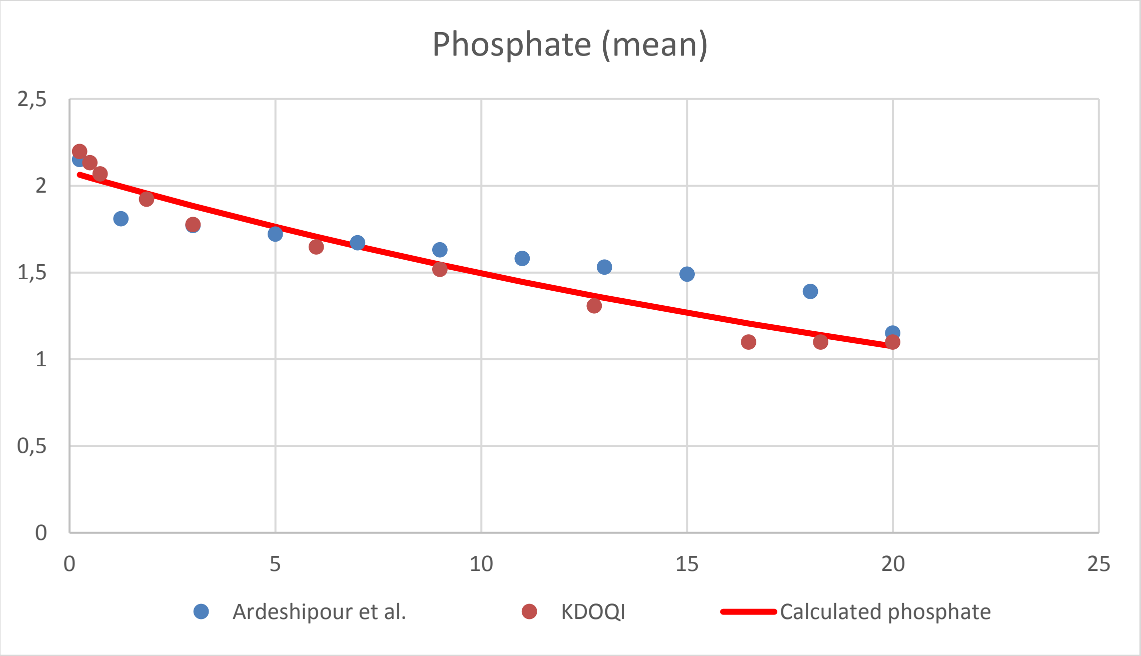

Alkaline phosphatase (asymmetric Gaussian model):

y = Intercept + B1*x^1 + B2*x^2 + B3*x^3 + B4*x^4

Alkaline phosphatase girls :

	-2SD	Mean	+2SD
Intercept	555.8029	289.3375	150.7132
B1	-141.594	-72.0826	-29.5663
B2	26.95152	16.80263	7.587733
B3	-1.81715	-1.36212	-0.68585
B4	0.03738	0.034009	0.018784

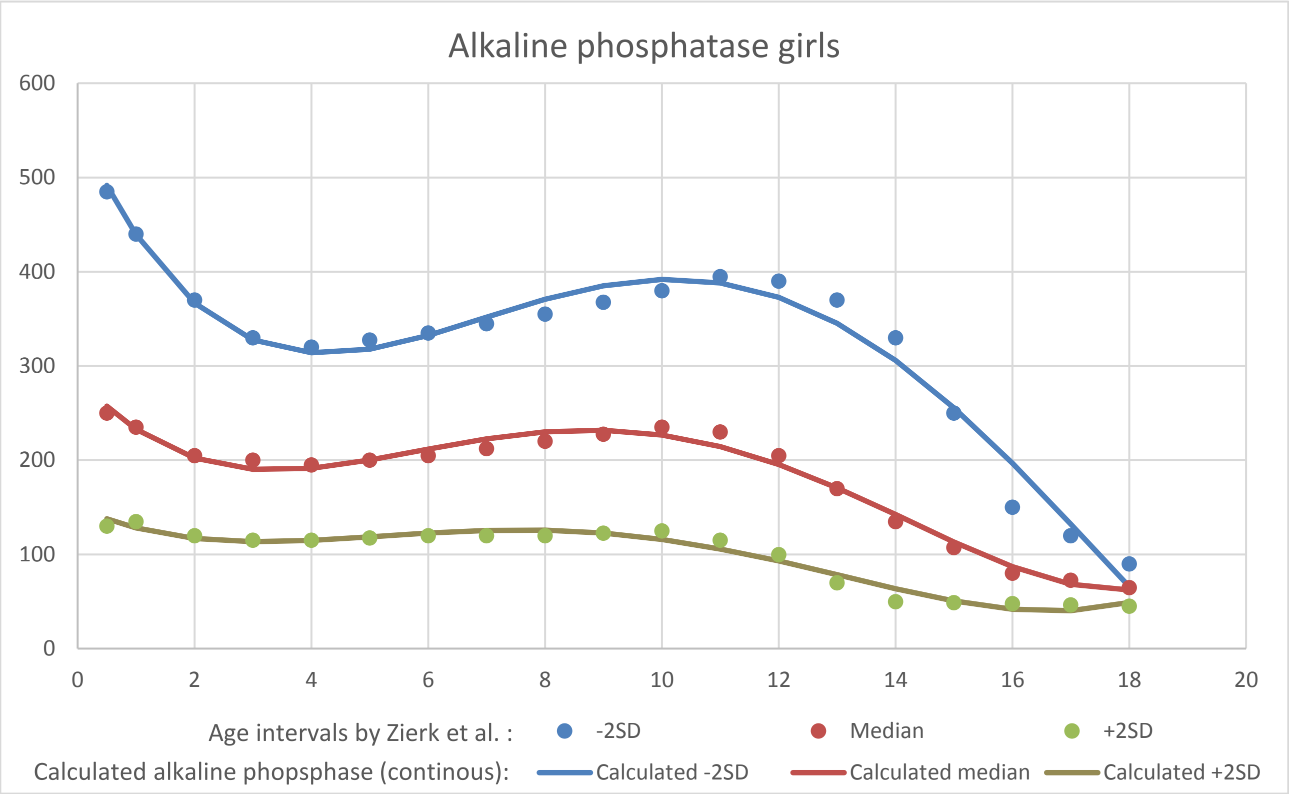

Alkaline phosphatase boys :

	-2SD	Mean	+2SD
Intercept	546.0365	280.9914	168.2496
B1	-113.846	-40.1935	-34.4237
B2	14.31947	5.392376	7.39377
B3	-0.38685	-0.16	-0.56229
B4	-0.00703	-0.00286	0.01317

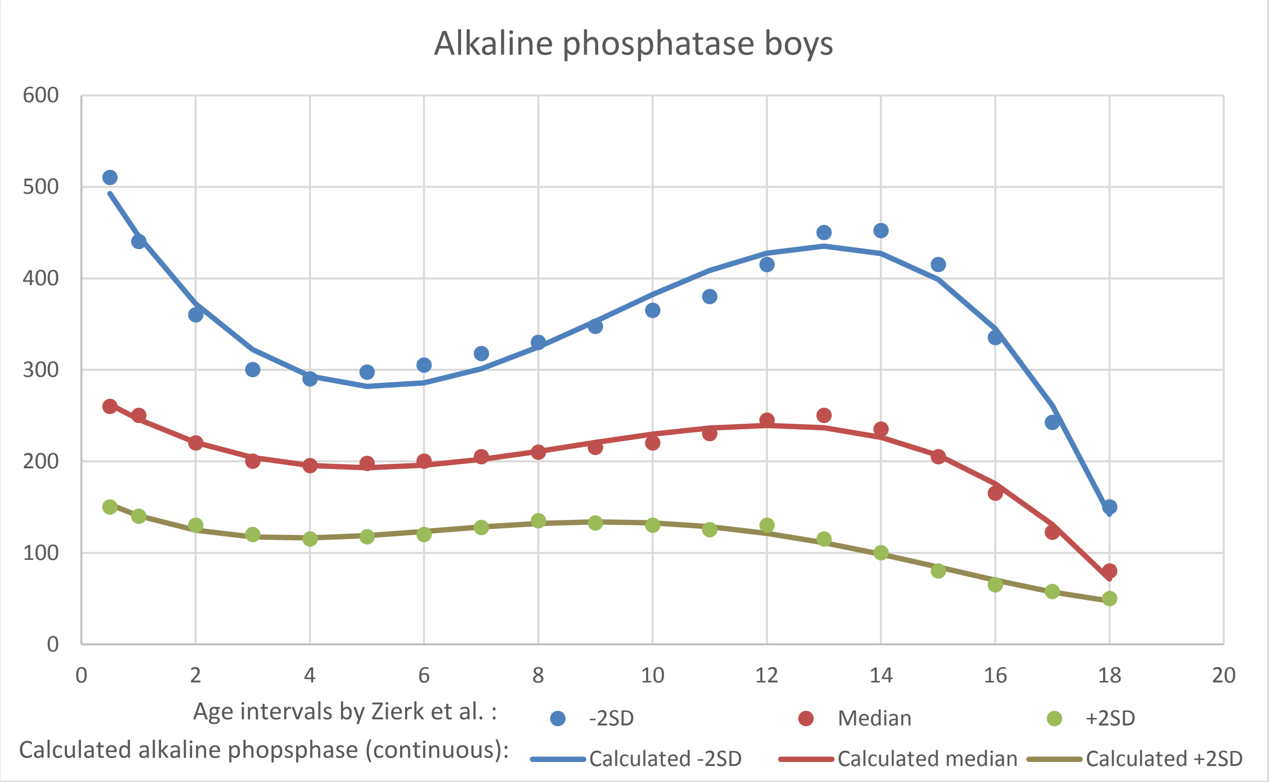

TmP/GFR-SDS (asymmetric Gaussian model):

y = A1*exp(-x/t1) + y0

	-2SD	Mean	+2SD
y0	0.652265	1.205662	1.655575
A1	0.65638	0.364298	0.376628
t1	5.577926	3.849471	0.8259

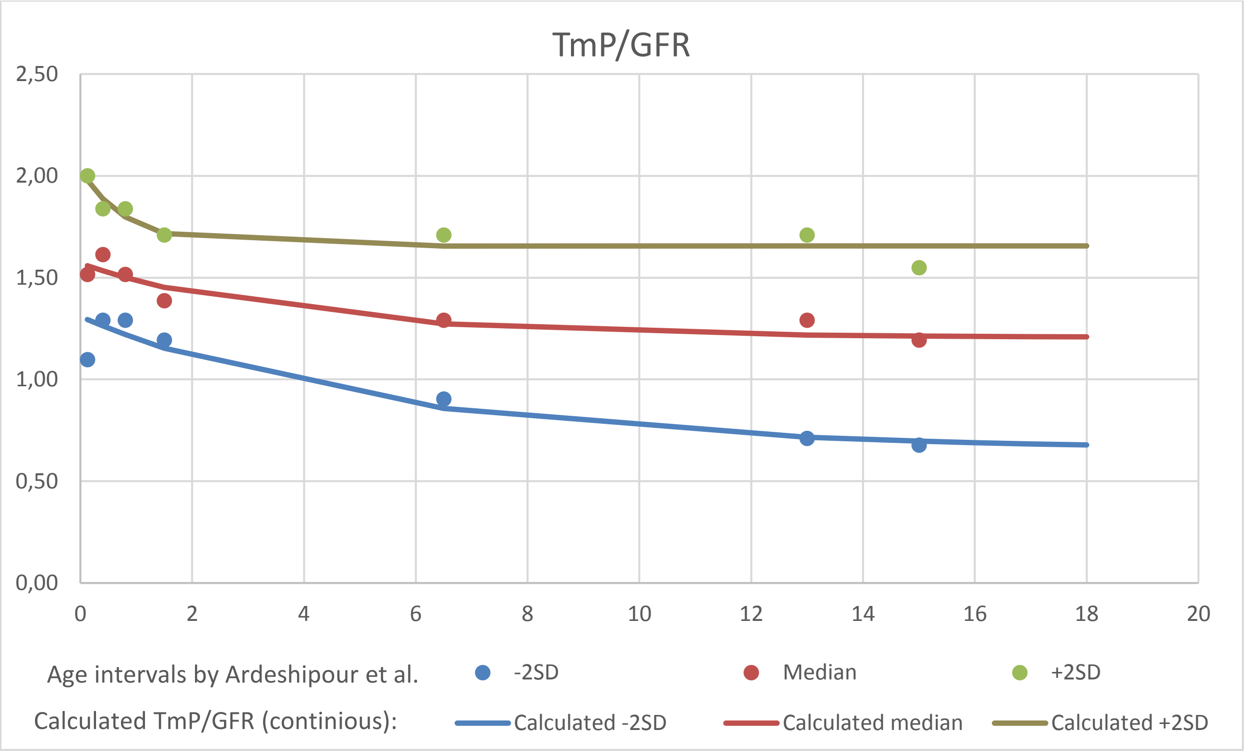

Supplemental tables

Supplemental table 1. Items of data collection form

**General data**	**If available, urine tests**
Sex	Calcium (if urinary creatinine available)
Month & year of birth (mm/yy)	Phosphate (if urinary creatinine available)
Month & year of test (mm/yy)	Creatinine
**Disease**	Calcium/creatinine
Bartter or Gitelman*	**Treatment (mandatory)**
Nephrocalcinosis (yes/no)	Indomethacin (yes/no)
**Mandatory serum data**	Ibuprophen (yes/no)
Creatinine	Other NSAIDS (yes/no)
Na (mEq/L)	Amilorid (yes/no)
K (mEq/L)	Triamterene (yes/no)
Cl (mEq/L)	Eplirenone (yes/no)
HCO3 (mEq/L)	KCl supplements (yes/no)
Phosphate	NaCl supplements (yes/no)
Calcium	Magnesium supplements (yes/no)
Magnesium	Proton pump inhibitors (yes/no)
iPTH (pg/ml)	Other gastric protectors (yes/no)
**Other non-mandatory blood tests**	Oral contraceptives (yes/no)
Uric acid	Vitamin D supplements (yes/no)
Alkaline phosphatase (IU/l)	Other relevant treatments (specify)
Total protein	
Albumin	
25OH vit D (nmol/L)	
(c-)FGF23 (RU/ml)	

* Options: Bartter I, Bartter II, Bartter III, Bartter IV, Bartter V, Gitelman; if genetic test is NOT available, please select “Bartter NOS” or “Gitelman NOS”.

Supplemental table 2. Valid variables and missing values

Variables	Bartter type I & II		Bartter type III		Bartter type NOS		Gitelman syndrome		Total	
	Valid N	Missing N	Valid N	Missing N	Valid N	Missing N	Valid N	Missing N	Valid N	Missing N
Characteristics										
Male : Female ratio, *n* (%)	107	0	88	0	90	0	304	0	589	0
Age (years)	107	0	88	0	90	0	304	0	589	0
Nephrocalcinosis, *n* (%)	101	6	88	0	90	0	304	0	525	64
Laboratory results										
iPTH (pmol/L)	107	0	88	0	90	0	304	0	589	0
Creatinine (umol/L)	107	0	88	0	90	0	304	0	589	0
Sodium (mmol/L)	104	3	88	0	89	1	304	0	585	4
Potassium (mmol/L)	107	0	88	0	89	1	304	0	588	1
Chloride (mmol/L)	98	9	85	3	81	9	283	21	547	42
Bicarbonate (mmol/L)	99	8	86	2	89	1	281	23	555	34
Phosphate (mmol/L)	104	3	88	0	90	0	257	47	539	50
Calcium (mmol/L)	105	2	88	0	90	0	303	1	586	3
Magnesium (mmol/L)	93	14	85	3	89	1	299	5	566	23
Uric acid (µmol/l)	56	51	67	21	70	20	124	180	317	272
Alkaline phosphatase (IU/L)	82	25	66	22	75	15	214	90	437	152
Total protein (g/L)	48	59	45	43	57	33	171	133	321	268
Albumin (g/L)	75	32	62	26	74	16	133	171	344	245
25OH vitamin D (nmol/L)	60	47	49	39	63	27	194	110	366	223
Urinary calcium/creatinine (mmol/mmol)	82	25	80	8	72	18	227	77	461	128
TRP / TmP/GFR	17	90	41	47	39	51	110	194	207	382
Treatment										
NSAIDs, *n* (%)										

Indomethacin, *n* (%)	107	0	88	0	90	0	304	0	589	0
Ibuprophen, *n* (%)	107	0	88	0	90	0	304	0	589	0
Other NSAID, *n* (%)	107	0	88	0	90	0	304	0	589	0
Potassium-sparing diuretics, *n* (%)										
Amilorid, *n* (%)	107	0	88	0	90	0	304	0	589	0
Triamterene, *n* (%)	107	0	88	0	90	0	304	0	589	0
Aldosterone antagonists, *n* (%)										
Eplerenone, *n* (%)	107	0	88	0	90	0	304	0	589	0
Spironolactone, *n* (%)	107	0	88	0	90	0	304	0	589	0
Canrenone, *n* (%)	107	0	88	0	90	0	304	0	589	0
ACE inhibitors/ARBs, *n* (%)	107	0	88	0	90	0	304	0	589	0
Hydrochlorothiazide, *n* (%)	107	0	88	0	90	0	304	0	589	0
Potassium supplements, *n* (%)	107	0	88	0	90	0	304	0	589	0
Sodium supplements, *n* (%)	107	0	88	0	90	0	304	0	589	0
Magnesium supplements, *n* (%)	107	0	88	0	90	0	304	0	589	0
Phosphate supplements, *n* (%)	107	0	88	0	90	0	304	0	589	0
Proton pump inhibitors, *n* (%)	107	0	88	0	90	0	304	0	589	0
Other gastric protectors, *n* (%)	107	0	88	0	90	0	304	0	589	0
Oral contraceptives, *n* (%)	107	0	88	0	90	0	304	0	589	0
Vitamin D supplements, *n* (%)	107	0	88	0	90	0	304	0	589	0

iPTH = intact parathyroid hormone; 25OH vitamin D = 25-hydroxy vitamin D; TRP = tubular reabsorption of phosphate; TmP/GFR = ratio of tubular maximum reabsorption of phosphate to GFR; NSAIDs = nonsteroidal anti-inflammatory drugs; ACE inhibitors = angiotensin converting enzyme inhibitors; ARBs = angiotensin II receptor blockers

Supplemental table 3. Correlation coefficients of age in all patients

Variable	N	r_s	p-value
iPTH	589	-0.245	< 0.001
Creatinine	589	0.600	< 0.001
Sodium	585	0.034	0.406
Potassium	588	-0.258	< 0.001
Chloride	547	0.013	0.758
Bicarbonate	555	0.236	< 0.001
Phosphate	539	-0.654	< 0.001
Phosphate-SDS	539	0.305	< 0.001
Calcium	586	-0.432	< 0.001
Magnesium	566	-0.475	< 0.001
Uric acid	317	0.265	< 0.001
Alkaline phosphatase	437	-0.795	< 0.001
Alkaline phosphatase-SDS	437	-0.472	< 0.001
Total protein	321	-0.146	0.009
Albumin	344	0.042	0.434
25OH vitamin D	366	0.211	< 0.001
Urinary calcium/creatinine ratio	461	-0.396	< 0.001
TRP	207	0.036	0.609
TmP/GFR	207	-0.531	< 0.001
TmP/GFR-SDS	207	-0.253	< 0.001

iPTH = intact parathyroid hormone; phosphate-SDS = age-related phosphate standard deviation score; alkaline phosphatase-SDS = age-related alkaline phosphatase standard deviation score; 25OH vitamin D = 25-hydroxy vitamin D; TRP = tubular reabsorption of phosphate; TmP/GFR = ratio of tubular maximum reabsorption of phosphate to GFR; TmP/GFR-SDS: age-related TmP/GFR standard deviation score;
r_s = Spearman's rank correlation coefficient

Supplemental table 4. Correlation coefficients of age in patients with Bartter syndrome type I & II

Variable	N	r_s	p-value
iPTH	107	0.116	0.236
Creatinine	107	0.772	< 0.001
Sodium	104	0.180	0.067
Potassium	107	-0.284	0.003
Chloride	98	0.016	0.872
Bicarbonate	99	0.384	< 0.001
Phosphate	104	-0.655	< 0.001
Phosphate-SDS	104	0.329	0.001
Calcium	105	-0.514	< 0.001
Magnesium	93	-0.404	< 0.001
Uric acid	56	0.262	0.051
Alkaline phosphatase	82	-0.528	< 0.001
Alkaline phosphatase-SDS	82	0.020	0.862
Total protein	48	0.223	0.127
Albumin	75	0.112	0.337
25OH vitamin D	60	0.028	0.831
Urinary calcium/creatinine ratio	82	-0.485	< 0.001
TRP	17	0.176	0.498
TmP/GFR	17	-0.598	0.011
TmP/GFR-SDS	17	-0.345	0.176

iPTH = intact parathyroid hormone; phosphate-SDS = age-related phosphate standard deviation score; alkaline phosphatase-SDS = age-related alkaline phosphatase standard deviation score; 25OH vitamin D = 25-hydroxy vitamin D; TRP = tubular reabsorption of phosphate; TmP/GFR = ratio of tubular maximum reabsorption of phosphate to GFR; TmP/GFR-SDS: age-related TmP/GFR standard deviation score;
r_s = Spearman's rank correlation coefficient

Supplemental table 5. Prevalence of hypo- and hyperparathyroidism

	**Bartter syndrome type I & II** N = 107	**Bartter syndrome type III** N = 88	**Bartter syndrome NOS** N = 90	**Gitelman syndrome** N = 304	**Total** N = 589
Hypoparathyroidism	5 (4.7%)	9 (10.2%)	6 (6.7%)	61 (20.1%)	81 (13.8%)
Normal iPTH	42 (39.3%)	60 (68.2%)	51 (56.7%)	222 (73.0%)	375 (63.7%)
Hyperparathyroidism	60 (56.1%)	19 (21.6%)	33 (36.7%)	21 (6.9%)	113 (22.6%)

iPTH = intact PTH parathyroid hormone
Hypoparathyroidism: iPTH < 2 pmol/l; hyperparathyroidism: iPTH > 7.0 pmol/l

Supplemental table 6. Characteristics of all patients with and without hyperparathyroidism

Variable	Hyperparathyroidism	No hyperparathyroidism	p-value
Number of patients	N = 133 (23%)	N = 456 (77%)	
Characteristics			
Age (years)	12.1 [4.7 – 17.8]	18.9 [9.2 – 36.3]	< 0.001
Sex (male), *n* (%)	67 (50)	215 (47)	0.554
Bartter syndrome, *n* (%)	112 (84)	173 (38)	< 0.001
Bartter syndrome type I & II, *n* (%)	60 (45)	47 (10)	< 0.001
Bartter syndrome type III, *n* (%)	19 (14)	69 (15)	0.890
Bartter syndrome NOS, *n* (%)	33 (25)	57 (13)	0.001
Gitelman syndrome, *n* (%)	21 (16)	283 (62)	< 0.001
Nephrocalcinosis, *n* (%)	78/124 (63)	77/401 (19)	< 0.001
Laboratory results			
iPTH (pmol/l)	9.6 [8.0 – 13.1]	3.2 [2.3 – 4 .4]	NT
Creatinine (µmol/l)	56 [42 – 71]	55 [40 – 69]	0.371
Sodium (mmol/l)	140 [137 – 141]	139 [138 – 141]	0.812
Potassium (mmol/l)	3.4 [3.0 – 3.8]	3.2 [2.8 – 3.6]	< 0.001
Chloride (mmol/l)	98 [95 – 101]	98 [95 – 100]	0.662
Bicarbonate (mmol/l)	27.3 [25.3 – 30.0]	28.8 [27.0 – 31.0]	< 0.001
Phosphate-SDS	-0.80 [-1.81 – 0.41]	-0.94 [-1.84 – 0.11]	0.554
Calcium (mmol/l)	2.44 [2.33 – 2.52]	2.43 [2.34 – 2.54]	0.667
Magnesium (mmol/l)	0.84 [0.72 – 0.95]	0.68 [0.59 – 0.80]	< 0.001
Uric acid (µmol/l)	315 [240 – 409]	238 [184 – 320]	< 0.001
Alkaline phosphatase-SDS	0.88 [-0.13 – 1.81]	-0.47 [-1.38 – 0.47]	< 0.001
Total protein (g/l)	74 [71 – 78]	73 [69 – 77]	0.037
Albumin (g/l)	46 [43 – 48]	46 [43 – 49]	0.513
25OH vitamin D (nmol/l)	39 [27 – 64]	55 [29 – 83]	0.021
Urinary calcium/creatinine (mmol/mmol)	0.72 [0.18 – 1.67]	0.16 [0.06 – 0.48]	< 0.001
TRP	0.87 [0.79 – 0.91]	0.91 [0.86 – 0.95]	< 0.001
TmP/GFR-SDS	-0.77 [-1.98 – 0.18]	-0.86 [-1.47 – -0.03]	0.560
Treatment			
Indomethacin or other NSAID, *n* (%)	76 (57)	126 (28)	< 0.001
Potassium-sparing diuretics, *n* (%)	10 (8)	71 (16)	0.021
Aldosterone antagonists, *n* (%)	19 (14)	80 (18)	0.430
ACE inhibitors/ARBs, *n* (%)	5 (4)	10 (2)	0.347
Hydrochlorothiazide, *n* (%)	5 (4)	4 (1)	0.031
Potassium supplements, *n* (%)	109 (82)	401 (88)	0.083
Sodium supplements, *n* (%)	36 (27)	100 (22)	0.242
Magnesium supplements, *n* (%)	36 (27)	240 (53)	< 0.001
Phosphate supplements, *n* (%)	1 (1)	5 (1)	1.000
Proton pump inhibitors, *n* (%)	20 (15)	77 (17)	0.691
Other gastric protectors, *n* (%)	12 (9)	29 (6)	0.332
Oral contraceptives, *n* (%)	1 (1)	22 (5)	0.038
Vitamin D supplements, *n* (%)	42 (32)	62 (14)	< 0.001

iPTH = intact parathyroid hormone; phosphate-SDS = age-related phosphate standard deviation score; alkaline phosphatase-SDS = age-related alkaline phosphatase standard deviation score; 25OH vitamin D = 25-hydroxy vitamin D; TRP = tubular reabsorption of phosphate; TmP/GFR-SDS: age-related TmP/GFR standard deviation score; NSAID = nonsteroidal anti-inflammatory drugs; ACE inhibitors = angiotensin converting enzyme inhibitors; ARBs = angiotensin II receptor blockers; NT = not tested

Categorical data is presented as number and percent and was analyzed by Fisher's exact test. Continuous data is presented as median with interquartile range and was analyzed by Mann-Whitney U test.

Supplemental table 7. Correlation coefficients of iPTH in all patients

Variable	N	r_s	p-value
Sex (male)	589	0.039	0.345
Age	589	-0.245	< 0.001
Bartter/Gitelman syndrome	589	0.444	< 0.001
Bartter syndrome type I & II	589	0.377	< 0.001
Bartter syndrome type III	589	0.061	0.140
Bartter syndrome NOS	589	0.152	< 0.001
Gitelman syndrome	589	-0.444	< 0.001
Nephrocalcinosis	525	0.414	< 0.001
Creatinine	589	< 0.001	0.998
Sodium	585	-0.001	0.978
Potassium	588	0.193	< 0.001
Chloride	547	-0.031	0.463
Bicarbonate	555	-0.118	0.005
Phosphate-SDS	539	0.005	0.913
Calcium	586	-0.016	0.707
Magnesium	566	0.387	< 0.001
Uric acid	317	0.204	< 0.001
Alkaline phosphatase-SDS	437	0.372	<0.001
Total protein	321	0.089	0.111
Albumin	344	-0.035	0.514
25OH vitamin D	366	-0.186	< 0.001
Urinary calcium/creatinine ratio	461	0.277	< 0.001
TRP	207	-0.276	< 0.001
TmP/GFR-SDS	207	0.016	0.818
Indomethacin or other NSAID	589	0.283	< 0.001
Potassium-sparing diuretics	589	-0.103	0.012
Aldosterone antagonists	589	-0.038	0.361
ACE inhibitors/ARBs	589	-0.020	0.622
Hydrochlorothiazide	589	0.078	0.057
Potassium supplements	589	-0.070	0.092
Sodium supplements	589	0.018	0.670
Magnesium supplements	589	-0.288	< 0.001
Phosphate supplements	589	0.004	0.932
Proton pump inhibitors	589	0.046	0.265
Other gastric protectors	589	0.027	0.521
Oral contraceptives	589	-0.081	0.048
Vitamin D supplements	589	0.171	< 0.001

iPTH = intact parathyroid hormone; phosphate-SDS = age-related phosphate standard deviation score; alkaline phosphatase-SDS = age-related alkaline phosphatase standard deviation score; 25OH vitamin D = 25-hydroxy vitamin D; TRP = tubular reabsorption of phosphate; TmP/GFR-SDS: age-related TmP/GFR standard deviation score; NSAID = nonsteroidal anti-inflammatory drug; ACE inhibitors = angiotensin converting enzyme inhibitors; ARBs = angiotensin II receptor blockers.

r_s = Spearman's rank correlation coefficient

Supplemental table 8. Multivariable logistic regression of hyperparathyroidism in all patients

Variable	Units	N	OR	95% C.I.			p-value
Disease	Bartter I & II	395	3.20	1.68	-	6.08	< 0.001
Calcium	mmol/l	395	0.03	0.01	-	0.21	< 0.001
Magnesium	mmol/l	395	18.47	2.67	-	127.93	0.003
Phosphate-SDS	mmol/l	395	1.08	0.92	-	1.27	0.343
Alkaline phosphatase-SDS	IU/l	395	1.26	1.11	-	1.43	< 0.001
Vitamin D supplements	Yes	395	1.87	0.99	-	3.52	0.052
Indomethacin or other NSAID	Yes	395	1.88	1.08	-	3.28	0.026

Phosphate-SDS = age-related phosphate standard deviation score; alkaline phosphatase-SDS = age-related alkaline phosphatase standard deviation score; NSAID = nonsteroidal anti-inflammatory drug

Supplemental table 9. Prevalence of hypo- and hyperphosphatemia

	**Bartter syndrome type I & II** N = 104	**Bartter syndrome type III** N = 88	**Bartter syndrome NOS** N = 90	**Gitelman syndrome** N = 257	**Total** N = 539
Hypophosphatemia	20 (19.2%)	28 (31.8%)	26 (28.9%)	42 (16.3%)	116 (21.5%)
Normal phosphate	76 (73.1%)	58 (65.9%)	62 (68.9%)	205 (79.8%)	401 (74.4%)
Hyperphosphatemia	8 (7.7%)	2 (2.3%)	2 (2.2%)	10 (3.9%)	22 (4.1%)

Age-related phosphate standard deviation score (phosphate-SDS) was used to determine prevalence of hypo- and hyperphosphatemia. Hypophosphatemia: phosphate-SDS < -2; hyperphosphatemia: phosphate-SDS > 2.

Supplemental table 10. Characteristics of all patients with and without hypophosphatemia

Variable	Hypophosphatemia	No hypophosphatemia	p-value
Number of patients	116 (21.5 %)	423 (78.5 %)	
Characteristics			
Age (years)	6.7 [2.18 – 19.9]	16.8 [9.9 – 34.0]	< 0.001
Sex (male), *n* (%)	60 (51.7%)	197 (46.6%)	0.346
Bartter syndrome, *n* (%)	74 (63.8%)	208 (49.2%)	0.006
Bartter syndrome type I & II, *n* (%)	20 (17.2%)	84 (19.9%)	0.596
Bartter syndrome type III, *n* (%)	28 (24.1%)	60 (14.2%)	0.015
Bartter syndrome NOS, *n* (%)	26 (22.4%)	64 (15.1%)	0.068
Gitelman syndrome, *n* (%)	42 (36.2%)	215 (50%)	0.006
Nephrocalcinosis, *n* (%)	39 (38.6%)	114 (30.4%)	0.120
Laboratory results			
Phosphate-SDS	-2.97 [-3.54 – -2.35]	-0.44 [-1.20 – 0.47]	NT
iPTH (pmol/l)	4.1 [2.6 – 7.0]	3.8 [2.5 – 6.9]	0.997
Creatinine (µmol/l)	44 [28 – 66]	55 [42 – 70]	< 0.001
Sodium (mmol/l)	139 [137 – 140]	140 [138 – 141]	0.003
Potassium (mmol/l)	3.3 [2.8 – 3.8]	3.3 [2.9 – 3.7]	0.844
Chloride (mmol/l)	98 [95 – 100]	98 [95 – 100]	0.736
Bicarbonate (mmol/l)	27.9 [25.7 – 30.3]	28.1 [26.2 – 30.7]	0.298
Calcium (mmol/l)	2.48 [2.41 – 2.58]	2.44 [2.34 – 2.53]	0.002
Magnesium (mmol/l)	0.76 [0.64 – 0.84]	0.72 [0.62 – 0.84]	0.121
Uric acid (µmol/l)	214 [161 – 302]	280 [209 – 369]	< 0.001
Alkaline phosphatase-SDS	0.22 [-0.73 – 1.10]	-0.07 [-1.16 – 0.96]	0.109
Total protein (g/l)	73 [69 – 76]	74 [70 – 77]	0.139
Albumin (g/l)	45 [42 – 47]	46 [44 – 49]	0.014
25OH vitamin D (nmol/l)	49 [33 – 84]	46 [27 – 78]	0.135
Urinary calcium/creatinine (mmol/mmol)	0.42 [0.11 – 1.41]	0.22 [0.07 – 0.75]	0.003
TRP	0.88 [0.83 – 0.93]	0.91 [0.86 – 0.94]	0.043
TmP/GFR-SDS	-2.04 [-2.63 – -1 .42]	-0.56 [-1.23 – 0.12]	< 0.001
Treatment			
Indomethacin or other NSAID, *n* (%)	54 (46.6%)	140 (33.1%)	0.009
Potassium-sparing diuretics, *n* (%)	17 (14.7%)	59 (13.9%)	0.880
Aldosterone antagonists, *n* (%)	20 (17.2%)	72 (17.0%)	1.000
ACE inhibitors/ARBs, *n* (%)	8 (6.9%)	7 (1.7%)	0.006
Hydrochlorothiazide, *n* (%)	2 (1.7%)	6 (1.4%)	0.684
Potassium supplements, *n* (%)	104 (89.7%)	364 (86.1%)	0.355
Sodium supplements, *n* (%)	34 (29.3%)	93 (22.0%)	0.109
Magnesium supplements, *n* (%)	48 (41.4%)	200 (47.3%)	0.293
Phosphate supplements, *n* (%)	3 (2.6%)	3 (0.7%)	0.117
Proton pump inhibitors, *n* (%)	27 (23.3%)	63 (14.9%)	0.036
Other gastric protectors, *n* (%)	14 (12.1%)	26 (6.1%)	0.044
Oral contraceptives, *n* (%)	7 (6.0%)	15 (3.5%)	0.286
Vitamin D supplements, *n* (%)	24 (20.7%)	76 (18.0%)	0.502

iPTH = intact parathyroid hormone; phosphate-SDS = age-related phosphate standard deviation score; alkaline phosphatase-SDS = age-related alkaline phosphatase standard deviation score; 25OH vitamin D = 25-hydroxy vitamin D; TRP = tubular reabsorption of phosphate; TmP/GFR-SDS: age-related ratio of tubular maximum reabsorption of phosphate to GFR standard deviation score; NSAID = nonsteroidal anti-inflammatory drug; ACE inhibitors = angiotensin converting enzyme inhibitors; ARBs = angiotensin II receptor blockers; NT = not tested. Categorical data is presented as number and percent and was analyzed by Fisher's exact test. Continuous data is presented as median and interquartile range and was analyzed by Mann-Whitney U test.

Supplemental table 11. Correlation coefficients of phosphate-SDS in all patients

Variable	N	r_s	p-value
Sex (male)	539	-0.069	0.111
Bartter/Gitelman syndrome	539	-0.105	0.015
Bartter syndrome type I & II	539	0.064	0.140
Bartter syndrome type III	539	-0.121	0.005
Bartter syndrome NOS	539	-0.088	0.042
Gitelman syndrome	539	0.105	0.015
Nephrocalcinosis	476	-0.040	0.379
iPTH	539	0.005	0.913
Creatinine	539	0.196	< 0.001
Sodium	535	0.152	< 0.001
Potassium	538	0.017	0.692
Chloride	499	-0.032	0.471
Bicarbonate	507	0.035	0.433
Calcium	538	-0.186	< 0.001
Magnesium	517	-0.069	0.119
Uric acid	311	0.227	< 0.001
Alkaline phosphatase-SDS	403	-0.028	0.572
Total protein	283	0.057	0.338
Albumin	342	0.107	0.049
25OH vitamin D	323	-0.085	0.126
Urinary calcium/creatinine ratio	414	-0.116	0.018
TRP	207	0.201	0.004
TmP/GFR-SDS	207	0.699	< 0.001
Indomethacin or other NSAID	539	-0.073	0.092
Potassium-sparing diuretics	539	-0.043	0.322
Aldosterone antagonists	539	-0.006	0.880
ACE inhibitors/ARBs	539	-0.059	0.174
Hydrochlorothiazide	539	0.010	0.825
Potassium supplements	539	-0.021	0.633
Sodium supplements	539	-0.092	0.033
Magnesium supplements	539	0.065	0.132
Phosphate supplements	539	-0.103	0.016
Proton pump inhibitors	539	-0.100	0.020
Other gastric protectors	539	-0.065	0.131
Oral contraceptives	539	-0.056	0.194
Vitamin D supplements	539	-0.034	0.425

iPTH = intact parathyroid hormone; phosphate-SDS = age-related phosphate standard deviation score; alkaline phosphatase-SDS = age-related alkaline phosphatase standard deviation score; 25OH vitamin D = 25-hydroxy vitamin D; TRP = tubular reabsorption of phosphate; TmP/GFR-SDS: age-related ratio of tubular maximum reabsorption of phosphate to GFR standard deviation score; NSAID = nonsteroidal anti-inflammatory drug; ACE inhibitors = angiotensin converting enzyme inhibitors; ARBs = angiotensin II receptor blockers.

r_s = Spearman's rank correlation coefficient

Supplemental table 12. Prevalence of hypo- and hyperphosphatemia in adult patients only

	**Bartter syndrome type I & II** N = 15	**Bartter syndrome type III** N = 20	**Bartter syndrome NOS** N = 20	**Gitelman syndrome** N = 176	**Total** N = 231
Hypophosphatemia	1 (6.7%)	1 (5.0%)	4 (20.0%)	27 (15.3%)	33 (14.3%)
Normal phosphate	14 (93.3%)	19 (95.0%)	16 (80.0%)	148 (84.1%)	197 (85.3%)
Hyperphosphatemia	0 (0.0%)	0 (0.0%)	0 (0.0%)	1 (0.6%)	1 (0.4%)

Serum phosphate was used to determine prevalence of hypo- and hyperphosphatemia. Hypophosphatemia: phosphate < 0.80 mmol/l; hyperphosphatemia: phosphate > 1.50 mmol/l.

Supplemental table 13. Correlation coefficients of iPTH in patients with Gitelman syndrome

Variable	N	r_s	p-value
Sex (male)	304	0.032	0.573
Age	304	0.092	0.110
Nephrocalcinosis	304	0.123	0.049
Creatinine	304	0.007	0.905
Sodium	304	-0.800	0.163
Potassium	304	0.089	0.123
Chloride	283	-0.120	0.043
Bicarbonate	281	-0.025	0.677
Phosphate-SDS	257	-0.046	0.459
Calcium	303	-0.057	0.326
Magnesium	299	0.188	0.001
Uric acid	124	-0.015	0.866
Alkaline phosphatase-SDS	214	0.164	0.016
Total protein	171	-0.071	0.357
Albumin	133	-0.028	0.750
25OH vitamin D	194	-0.207	0.004
Urinary calcium/creatinine ratio	227	-0.078	0.243
TRP	110	-0.125	0.192
TmP/GFR-SDS	110	-0.099	0.302
Indomethacin or other NSAID	304	0.069	0.230
Potassium-sparing diuretics	304	0.018	0.761
Aldosterone antagonists	304	0.019	0.745
ACE inhibitors/ARBs	304	-0.087	0.131
Potassium supplements	304	0.051	0.379
Sodium supplements	304	-0.101	0.077
Magnesium supplements	304	-0.019	0.741
Phosphate supplements	304	-0.049	0.390
Proton pump inhibitors	304	0.063	0.273
Other gastric protectors	304	0.071	0.219
Oral contraceptives	304	-0.032	0.579
Vitamin D supplements	304	-0.089	0.120

iPTH = intact parathyroid hormone; phosphate-SDS = age-related phosphate standard deviation score; alkaline phosphatase-SDS = age-related alkaline phosphatase standard deviation score; 25OH vitamin D = 25-hydroxy vitamin D; TRP = tubular reabsorption of phosphate; TmP/GFR-SDS: age-related ratio of tubular maximum reabsorption of phosphate to GFR standard deviation score; NSAID = nonsteroidal anti-inflammatory drug; ACE inhibitors = angiotensin converting enzyme inhibitors; ARBs = angiotensin II receptor blockers.
r_s = Spearman's rank correlation coefficient

Supplemental figures

Supplemental figure 1. Scatterplots of phosphate and phosphate-SDS with age in all patients and patients with Bartter syndrome type I & II

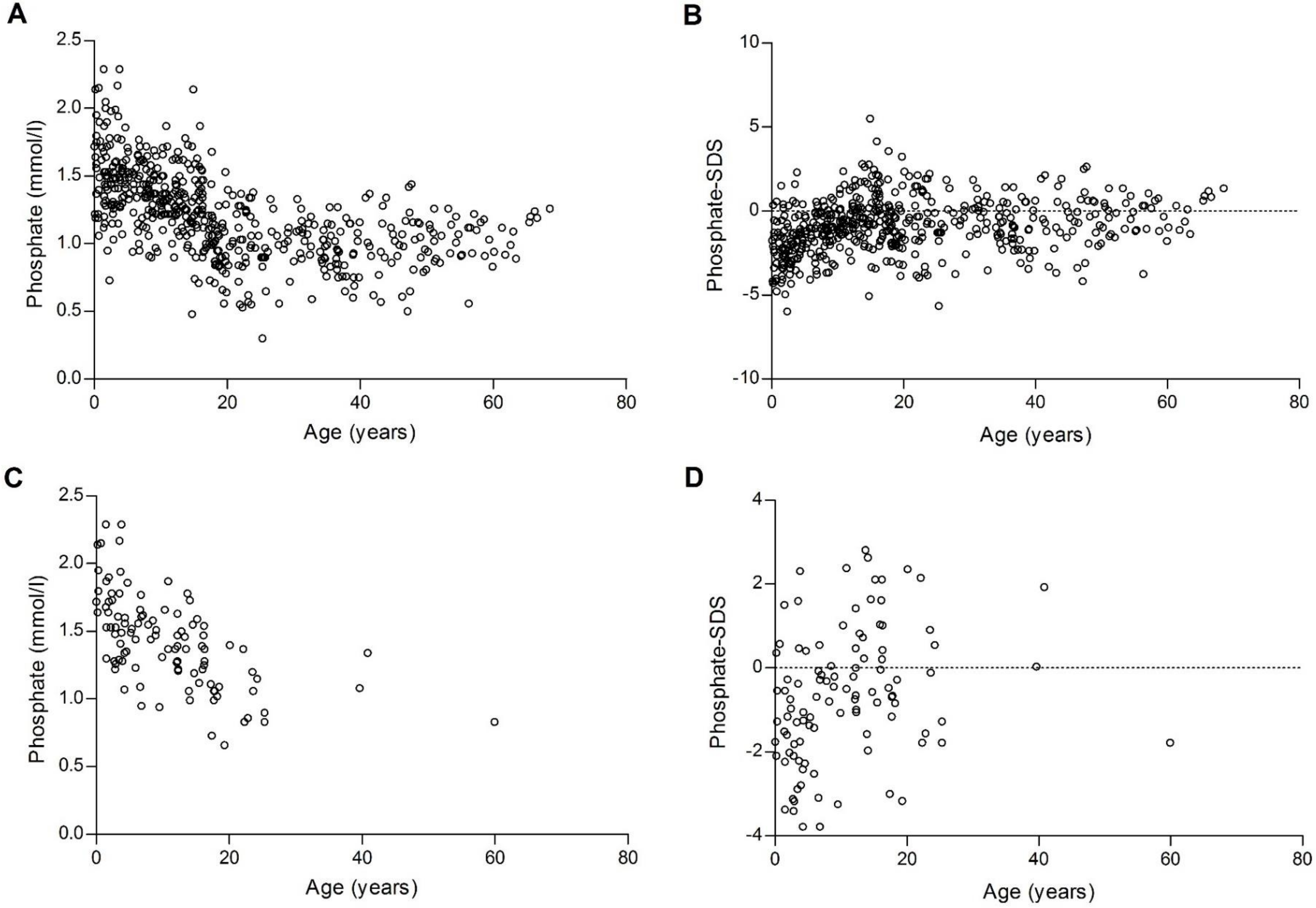

A. Scatterplot of age and phosphate in all patients. **B.** Scatterplot of age and phosphate-SDS in all patients. **C.** Scatterplot of age and phosphate in Bartter syndrome type I & II. **D.** Scatterplot of age and phosphate-SDS in Bartter syndrome type I & II.

Supplemental figure 2. Scatterplots of alkaline phosphatase and alkaline phosphatase-SDS in all patients and patients with Bartter syndrome type I & II

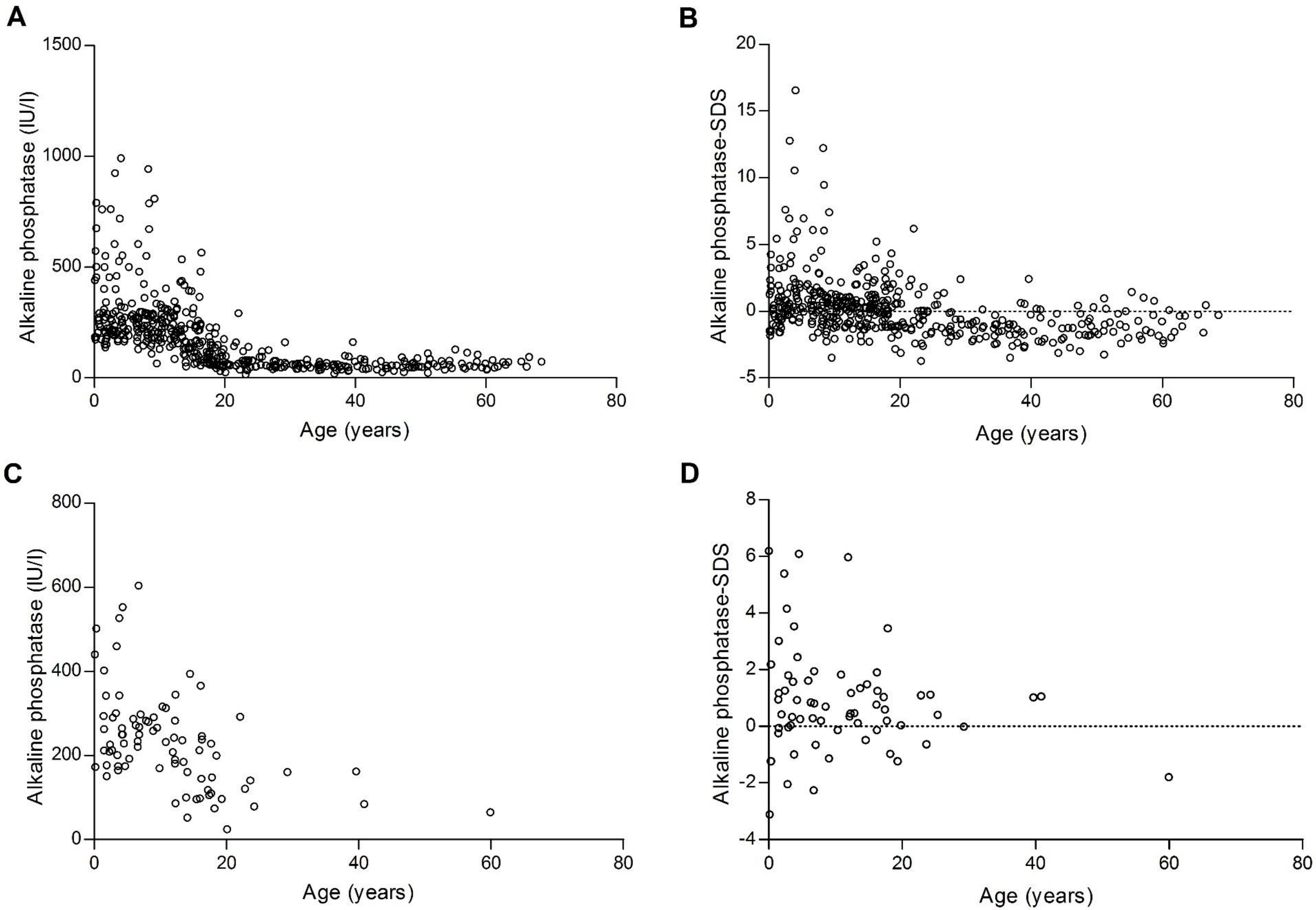

A. Scatterplot of age and alkaline phosphatase in all patients. **B.** Scatterplot of age and alkaline phosphatase-SDS in all patients. **C.** Scatterplot of age and alkaline phosphatase in Bartter syndrome type I & II. **D.** Scatterplot of age and alkaline phosphatase-SDS in Bartter syndrome type I & II.

Supplemental figure 3. Scatterplots of TmP/GFR and TmP/GFR-SDS in all patients

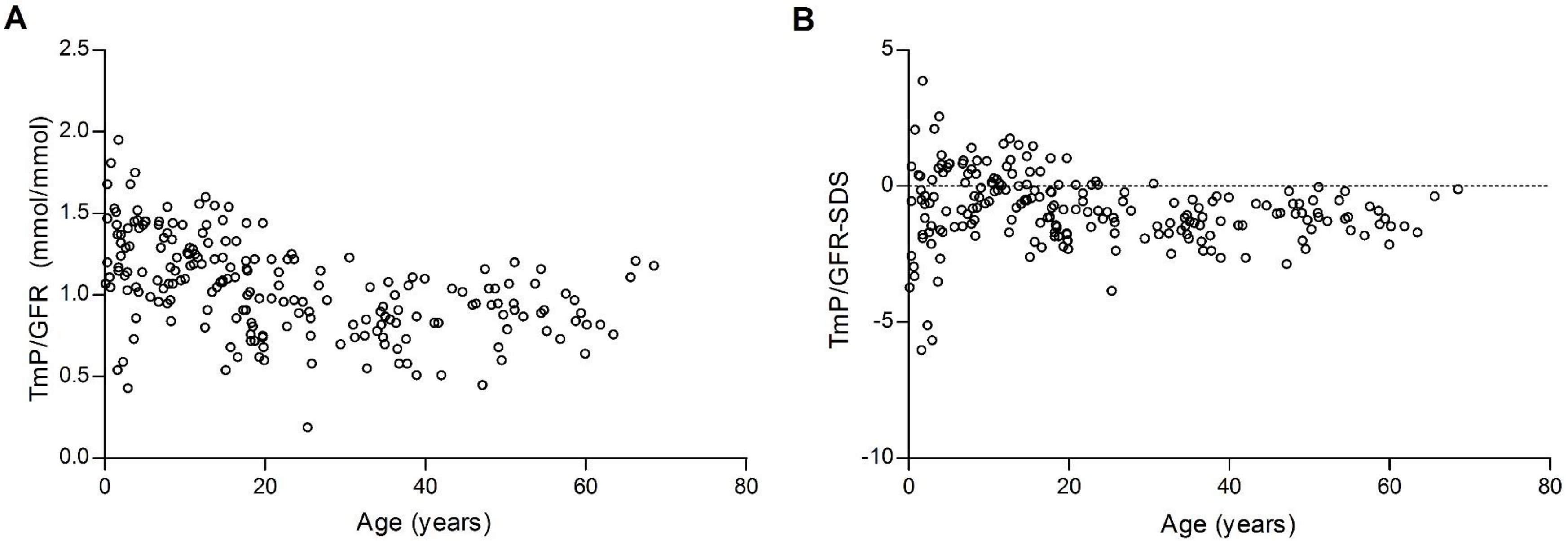

TmP/GFR-SDS: ratio of tubular maximum reabsorption of phosphate to GFR. TmP/GFR-SDS: age-related ratio of tubular maximum reabsorption of phosphate to GFR standard deviation score

A. Scatterplot of age and TmP-GFR in all patients. **B.** Scatterplot of age and TmP/GFR-SDS in all patients.
